# Repulsive segregation of fluoroalkyl side chains turns a cohesive polymer into a mechanically tough, ultrafast self-healable, nonsticky elastomer

**DOI:** 10.1038/s41598-022-16156-9

**Published:** 2022-07-25

**Authors:** Yohei Miwa, Taro Udagawa, Shoichi Kutsumizu

**Affiliations:** 1 Department of Chemistry and Biomolecular Science, Faculty of Engineering, Gifu University, Yanagido, Gifu, 501-1193 Japan; 2 PRESTO, Japan Science and Technology Agency, Kawaguchi, Japan

**Keywords:** Mechanical properties, Supramolecular polymers

## Abstract

Dynamic crosslinking of flexible polymer chains via attractive and reversible interactions is widely employed to obtain autonomously self-healable elastomers. However, this design leads to a trade-off relationship between the strength and self-healing speed of the material, *i.e*., strong crosslinks provide a mechanically strong elastomer with slow self-healing property. To address this issue, we report an “inversion” concept, in which attractive poly(ethyl acrylate-*random*-methyl acrylate) chains are dynamically crosslinked via repulsively segregated fluoroalkyl side chains attached along the main chain. The resulting elastomer self-heals rapidly (> 90% within 15 min) via weak but abundant van der Waals interactions among matrix polymers, while the dynamic crosslinking provides high fracture stress (≈2 MPa) and good toughness (≈17 MJ m^−3^). The elastomer has a nonsticky surface and selectively self-heals only at the damaged faces due to the surface segregation of the fluoroalkyl chains. Moreover, our elastomer strongly adheres to polytetrafluoroethylene plates (**≈**60 N cm^−2^) via hot pressing.

## Introduction

Since the discovery of polytetrafluoroethylene (PTFE) in 1938, fluorinated polymers have contributed greatly to our daily lives due to their outstanding properties such as nonstick and antiadhesive properties, chemical and thermal stabilities, low friction coefficients, biocompatibility, and extremely low surface tension^1^. For example, coating frying pans with Teflon^®^ has facilitated daily cooking because it provides nonsticky and water-and-oil repellent surfaces. As another example of fluorinated polymers, fluoroelastomers with enhanced heat and chemical stabilities and durability stemming from the presence of fluorine are essential in the automotive, aviation, and aerospace industries^2^. Here, we report a novel application of the fluorine component: A rapid self-healable and mechanically tough elastomer crosslinked via segregation of fluoroalkyls has been developed.

Considering the tremendous amount of elastomers that are used in various products, such as vehicles, electrical appliances, medical and cosmetic products, and buildings, the development of self-healing elastomers to improve significantly the lifetime, safety, energy efficiency, and mechanical toughness of the products attracts increasing research attention^3–5^. Furthermore, self-healing elastomers can be expected to provide access to futuristic high-performance products such as artificial skin, soft robots, wearable sensors and PCs, and smart actuators^6–8^.

Research in this regard has led to the development of many self-healing elastomers with various healing mechanisms. One of the most common approaches to obtain self-healing elastomers is the physical crosslinking of flexible polymers with low glass transition temperature (*T*_g_) via attractive and reversible interactions such as hydrogen bonds^9–17^, dynamic covalent bonds^18–24^, host–guest interactions^25–28^, metal–ligand coordination^29–37^, and ionic bonds and associations^38–44^. These elastomers self-heal due to the reversibility of the physical crosslinks at the damaged faces. By moderately tuning the strength of the physical crosslinks, elastomers can autonomously self-heal at ambient temperature without requiring healing agents or external stimuli^10–12,15–17,21–24,26–28,30–44^. Such elastomers are more practical to produce maintenance-free products. However, their design encounters a critical problem: a trade-off relationship between the strength and the autonomous healing speed of the material. This implies that stronger physical crosslinks provide stiffer and stronger materials, but the self-healing speed decreases because of the presence of less rearranging networks at the damaged faces. In such conventional designs, both the self-healing property and the strength depend on the exchangeable crosslinks, which is the main cause for the trade-off relationship.

Herein, we report a novel design to enhance both the self-healing speed and strength of a thermoplastic elastomer. Our key concept is the inversion of the conventional design, in which flexible polymer chains are physically crosslinked via attractive bonds or interactions. In contrast, in our design, attractive polymer chains are physically crosslinked via repulsively segregated fluoroalkyl side chains attached along the main chain, which newly utilizes the fluorine component of fluoroelastomers. In the resulting elastomer, the strength is enhanced by the physical crosslinks via repulsive segregation, while the self-healing property is realized through the cohesion between the attractive polymer matrices between the damaged faces. Such attractive self-healing via van der Waals interactions have been recently reported in acrylic-based^45–48^ and polystyrene-*block*-polybutadiene-*block*-polystyrene-based^49,50^ polymers. Importantly, upon moderately tuning the extent of segregation, the network can exhibit dynamic nature as in the conventional designs, which enhances the self-healing property and several mechanical functions such as the fracture and fatigue resistance of the material. As a result, a novel multifunctional, self-healing, fluorinated elastomer is obtained.

## Results

### Material design

**Figure 1:**
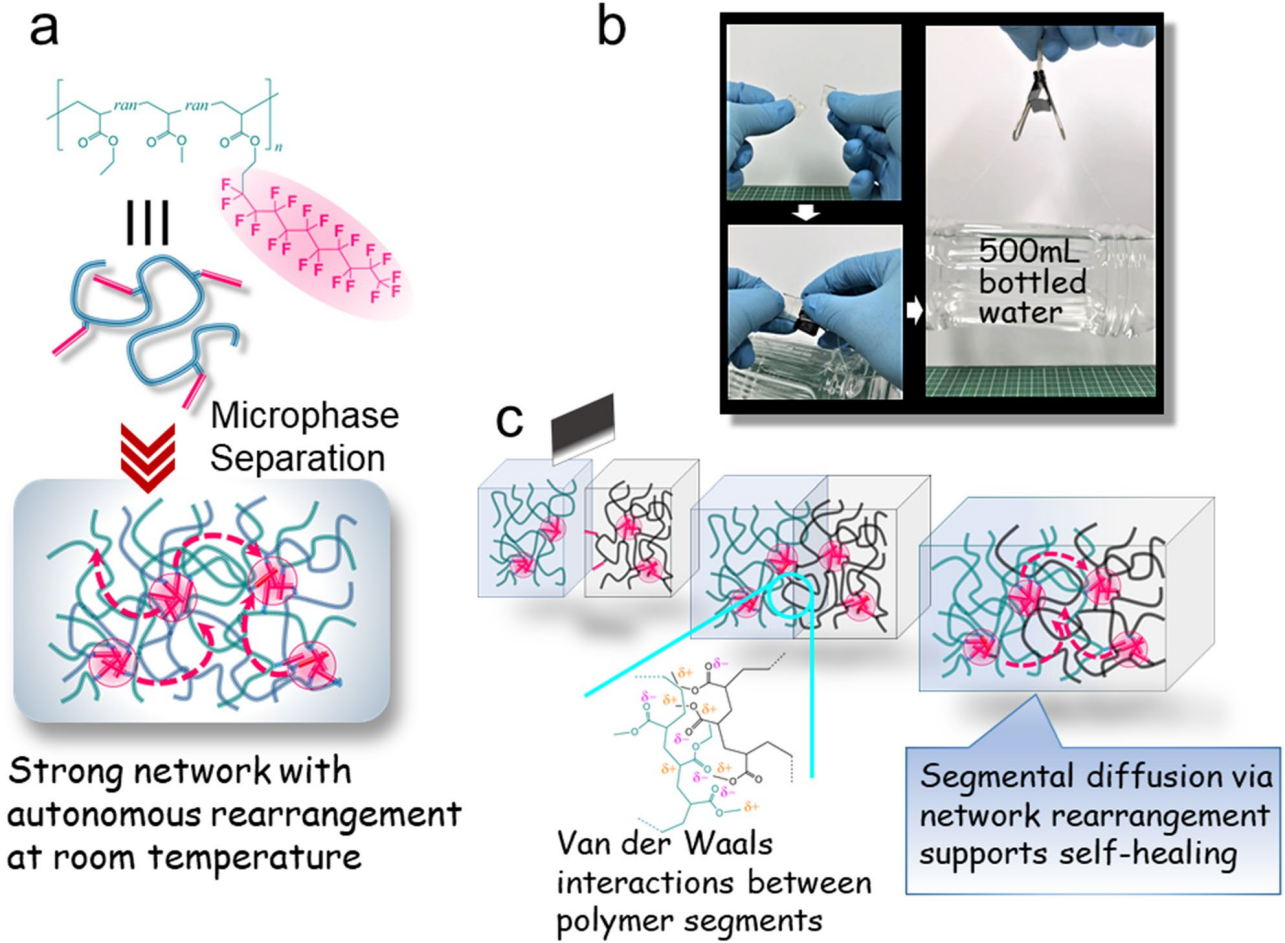
Molecular structure and self-healing mechanism for PEMA4F21. (**a**) Chemical structure and schematic illustration of the aggregation state of the fluorinated poly(ethyl acrylate-*random*-methyl acrylate) (PEMA) specimen PEMA4F21. (**b**) Photograph of the ultrafast self-healing behavior of a cut PEMA4F21 block. (**c**) Schematic illustration of the self-healing mechanism of PEMA4F21. PEMA segments cohere via weak but abundant intermolecular van der Waals interactions at the cut faces. The approach of PEMA segments is accelerated via segmental diffusion between the cut faces induced by the network rearrangement.

Figure 1a depicts a schematic illustration of our design, which is based on poly(ethyl acrylate-*random*-methyl acrylate) (PEMA) containing fluoroalkyl side chains. As density functional theory (DFT) calculations demonstrate, the PEMA segments are attractive to each other due to weak but abundant interchain van der Waals interactions, whereas the interaction between the fluoroalkyl side chains is very small (Supplementary Fig. 4). Nevertheless, the repulsive interaction between the PEMA and fluoroalkyl components results in a strong exclusive segregation of the fluoroalkyl side chains into nanosized domains in the PEMA matrix (Fig. 1a), affording a polymer network. Despite not being crosslinked via attractive interactions, the network significantly enhances the mechanical strength of the material which is discussed later. At the same time, this network autonomously and moderately rearranges at room temperature because of the hopping of fluoroalkyl side chains between the nanosized domains (Fig. 1a). This elastomer readily self-heals at ambient temperature (Fig. 1b and Supplementary Movie S1). This ultrarapid healing occurs due to the cohesion between the attractive PEMA segments at the damaged faces (Fig. 1c), which is accelerated by the segmental diffusion of the PEMA segments between damaged faces induced by the network rearrangement. Here it should be emphasized that both the mechanical strength and the self-healing speed are enhanced by the action of the dynamic crosslinks, unlike in conventional self-healing elastomers.

The fluoroalkyl components impart several practical functions to the elastomer, i.e., a nonsticky surface due to the surface segregation of the fluoroalkyl component and the selective self-healing at the damaged faces thanks to this surface protection (Movie S1). From the viewpoint of practical applications, this property is important to prevent undesirable fusion between the elastomer surfaces.

### Materials synthesis and characterizations

We introduced relatively long fluoroalkyl side chains with 17, 21, or 23 fluorine atoms along the PEMA main chain via radical copolymerization (Fig. 1a and Supplementary Fig. 1). The obtained fluorinated PEMAs are denoted as PEMA*x*F*y*, where *x* and *y* represent the fluoroalkyl content in mol% and the number of fluorine atoms in a fluoroalkyl side chain, respectively. For comparative purposes, we also synthesized PMA4F21 and PEA4F21 having poly(methyl acrylate) (PMA) and poly(ethyl acrylate) (PEA) main chains, respectively, and a 1H,1H,2H,2H-perfluorododecyl acrylate homopolymer denoted as PF21. We characterized the materials by gel permeation chromatography (GPC), ^1^H nuclear magnetic resonance (NMR) (Supplementary Fig. 2), and Fourier transform infrared (FT-IR) spectroscopy (Supplementary Fig. 3). Table 1 summarizes the molecular characteristics of selected materials, and Supplementary Table 1 compiles other data.

**Table 1:** Summary of molecular characteristics of the studied samples.

Notation	*M*_w_^*a*^	*M*_w_/*M*_n_^*a*^	Fluoroalkyl unit^*b*^ /mol%	MA unit^*b*^/mol%	EA unit^*b*^/mol%	Fluoroalkylunit^*b*^/wt%	*R*_1_^c^/nm	*R*_CA_^c^/nm	*ND*^c^/(10 nm)^−3^
PEMA	425,200	6.72	N/A	45	55	N/A	N/A	N/A	N/A
PEMA4F21	379,000	4.11	4	45	51	22	1.56	2.32	13.7
PEMA4F17	249,500	5.04	4	44	52	19	1.29	1.99	10.6
PEMA7F23	133,600	2.19	7	30	63	35	1.61	2.30	18.4
PMA	235,700	3.51	N/A	100	N/A	N/A	N/A	N/A	N/A
PMA4F21	274,000	4.20	4	96	N/A	23	1.64	2.46	14.7
PEA	358,800	4.53	N/A	N/A	100	N/A	N/A	N/A	N/A
PEA4F21	248,900	3.97	4	N/A	96	21	1.50	2.28	10.6

^*a*^Weight average molecular weight (*M*_w_) and polydispersity index (*M*_w_/*M*_n_) were measured by GPC using polystyrene standards. ^b^The component was determined by ^1^H NMR. ^c^The radius (*R*_1_), half value of the closest approach limitation (*R*_CA_), and number density (*ND*) of fluoroalkyl domains were determined by SAXS.

**Figure 2:**
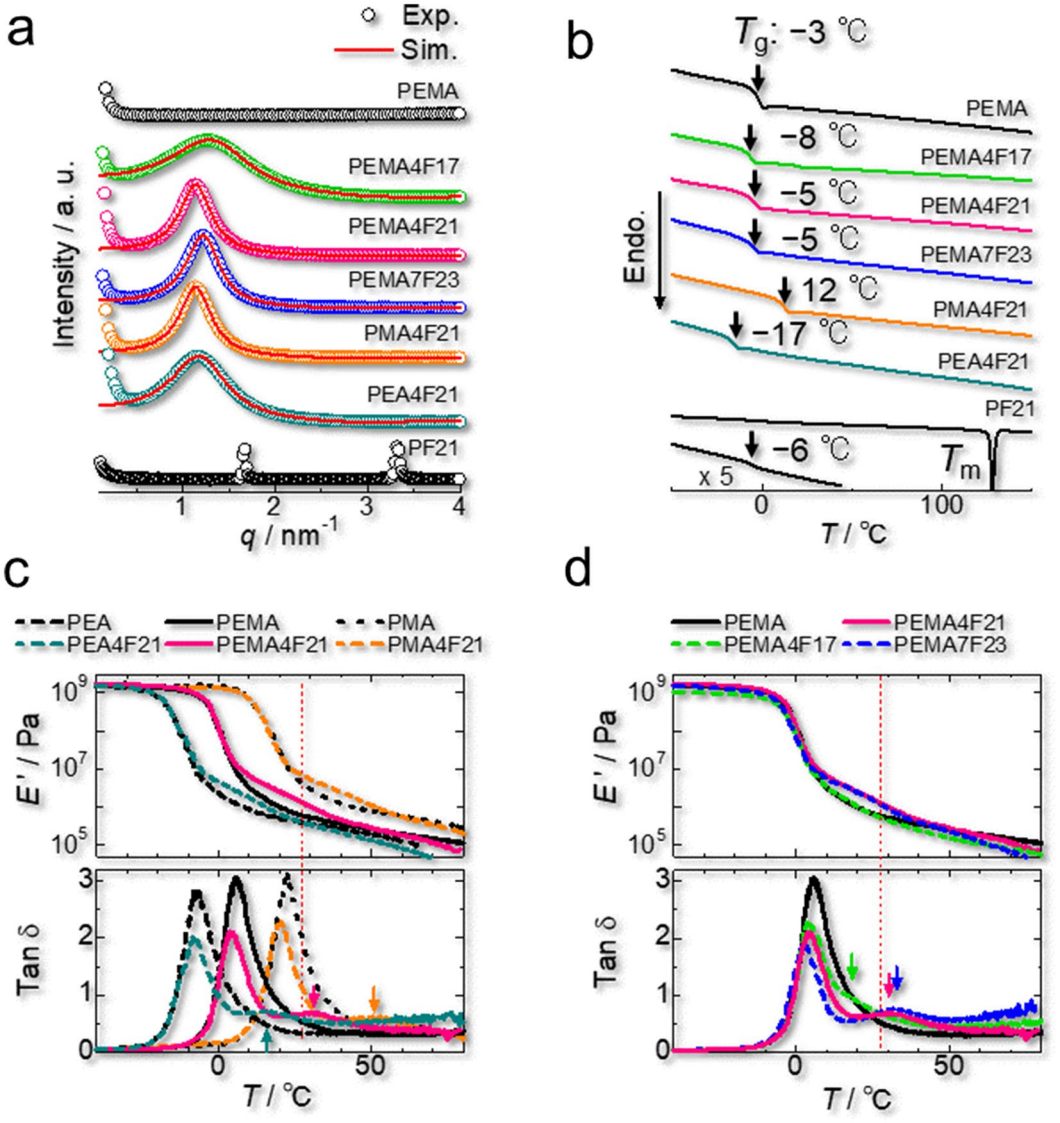
Effect of fluoroalkyl side chains on the structure and viscoelastic properties of the materials. (**a**) Experimental and simulated small-angle X-ray scattering patterns of the fluorinated elastomers. Simulations were performed according to the Yarusso–Cooper model^51^. (**b**) Differential scanning calorimetry traces of the fluorinated elastomers. The glass transition temperature (*T*_g_) of each polymer is indicated with an arrow. (**c**) Effect of fluoroalkyl side chains on the temperature dependence of the storage modulus (*E*') and tanδ for the indicated polymers. (**d**) Temperature dependence of *E*' and tanδ for fluorinated poly(ethyl acrylate-*random*-methyl acrylate) PEMA*x*F*y* having different fluoroalkyl side chain lengths.

As shown schematically in Fig. 1a, fluoroalkyl side chains are strongly segregated into nanosized domains in the polyacrylate matrix, forming a polymer network. The shape, size, and number density (*ND*) of the fluoroalkyl domains were determined by simulating a broad peak from the interference between fluoroalkyl domains in the small-angle X-ray scattering (SAXS) pattern (Fig. 2a and Supplementary Fig. 5). The Yarusso–Cooper model used for the simulation, which is described in the Supplementary Information in detail, assumes that spherical nanosized domains with a radius of *R*_1_ are randomly dispersed with the closest approach limitation (2*R*_CA_) in the matrix^51^. The *R*_1_, *R*_CA_, and *ND* values of the samples are listed in Table 1. The *R*_1_ value increases with increasing the length of the fluoroalkyl side chains, whereas it decreases with an increase in the cross-sectional area of the alkyl acrylate main chain because the steric hindrance of the main chain disturbs the segregation of fluoroalkyl side chains. The *ND* value increases with the weight fraction of fluoroalkyl side chains. Crystalline diffractions can be observed in the SAXS patterns of PEMA8F21, PEMA14F21, and PF21 with high fluoroalkyl content (Fig. 2a and Supplementary Fig. 5).

### Optimization of the polymer structure for dynamic crosslinks

To impart dynamic nature to the polymer network, the fluoroalkyl domains should be rubbery at room temperature because glassy and crystalline fluoroalkyl domains strictly restrict the polymer chain motion and hinder the network rearrangement. According to the differential scanning calorimetry (DSC) results (Fig. 2b), the present samples (except the pure homopolymer PF21) are amorphous. No melting transition and only a glass transition assigned to the matrix region was observed in the DSC measurements. X-ray diffraction further confirmed that no crystalline component was present in these materials (Supplementary Fig. 7). Meanwhile, the fluoroalkyl domains in the fluorinated samples in Fig. 2b can be expected to be rubbery at room temperature because PF21 exhibits a *T*_g_ of − 6 °C. Samples with higher fluoroalkyl contents exhibited a melting transition in their DSC results (Supplementary Fig. 6).

The dynamic mechanical analysis (DMA) results depicted in Fig. 2c and d reveal that due to the polymer network formed by the segregation of the fluoroalkyl component, PEMA4F21, PEMA7F23, PEA4F21, and PMA4F21 exhibit higher storage modulus (*E*') than the respective nonfluorinated polymers in the rubbery plateau region, whereas the relatively short fluoroalkyl side chains in PEMA4F17 do not form an elastic network. Such an elastic behavior is typically observed in transient networks, as was first theoretically studied by Leibler et al.^52^.

The relaxation temperatures defined as the tanδ peak for PEA4F21, PEMA4F21, PEMA7F23, and PMA4F21 are 16 °C, 31 °C, 33 °C, and 50 °C, respectively. This result indicates that the dissociation of the crosslinks occurs more easily in polymers with lower *T*_g_. Here, the *T*_g_ of PEMA4F21 is precisely tunable by adjusting the ratio of methyl acrylate to ethyl acrylate units along the chain. Moreover, an increase in the fluoroalkyl chain length restricts the dissociation (Fig. 2d). Additionally, an increased fluoroalkyl content restricts dissociation owing to the crystallization of fluoroalkyl domains (Supplementary Fig. 8). The dissociation of the crosslinks triggers the autonomous rearrangement of the polymer network because the detached fluoroalkyl side chains quickly segregate into other fluoroalkyl domains. That is, the “hopping” of fluoroalkyl side chains between domains results in the network rearrangement, as illustrated in Fig. 1a. A similar network rearrangement is typically observed for ionomers where hydrophobic polymer chains are physically crosslinked via attractive aggregation of hydrophilic ionic groups attached to the polymer chains^38–41,53,54^. As a result of the network rearrangement, some ionomers self-heal at room temperature^38,40–42^. One may think that the self-healing mechanism of our fluorinated elastomer is similar with ionomers. However, unlike our fluorinated elastomer, cut ionomers mainly self-heal by the interdiffusion of the attractive ionic groups between the damaged faces, which is a lengthy process requiring dozens of hours. Alternatively, our fluorinated elastomer self-heals via cohesion between attractive PEMA segments at the damaged faces, where the network rearrangement accelerates the approach between PEMA segments via segmental diffusion of the polymer chains (Fig. 1c).

### Mechanical studies

**Figure 3:**
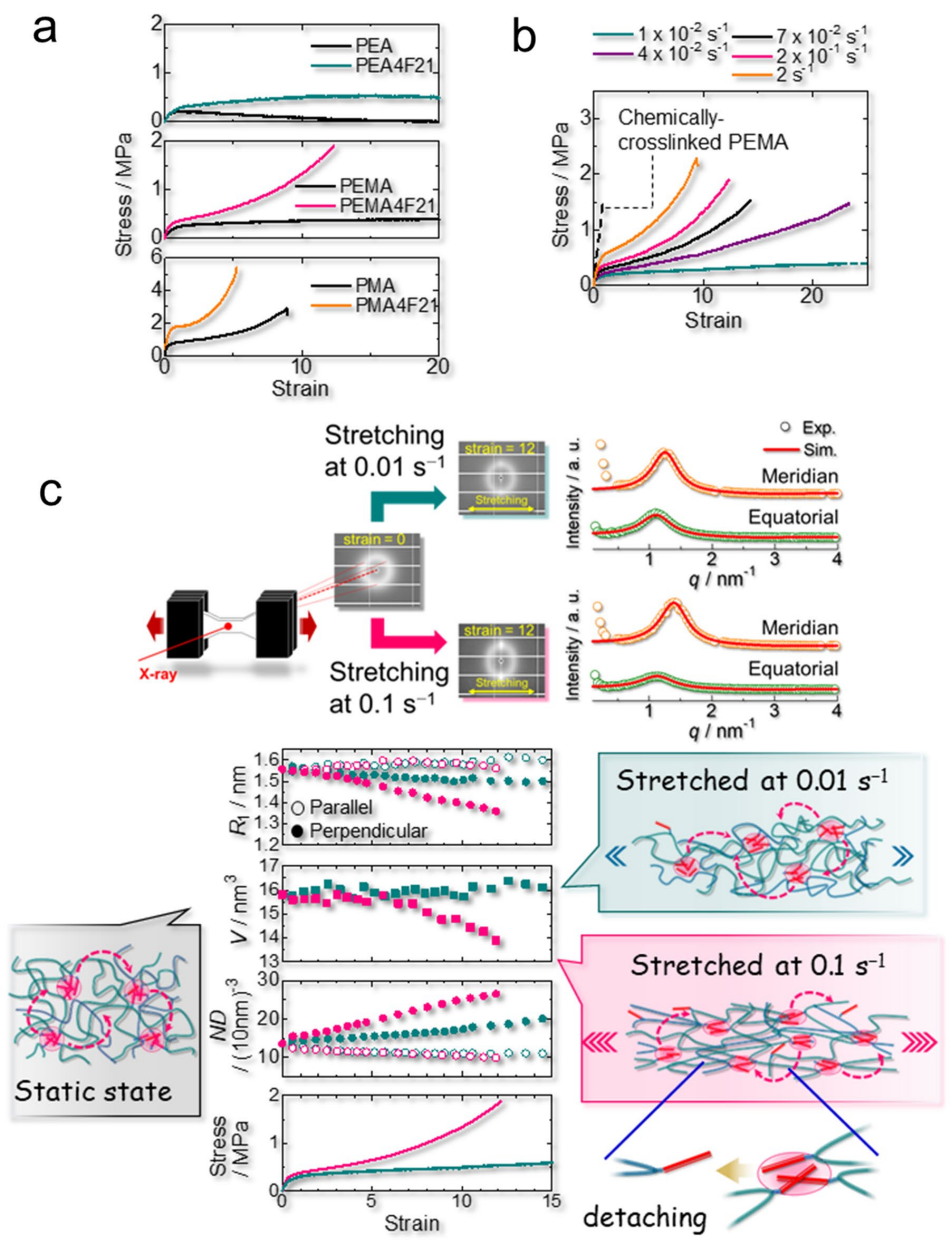
Effect of fluoroalkyl side chains on the tensile behavior of PEMA4F21. (**a**) Tensile tests for the indicated polymers measured at 27 °C ± 1 °C at a strain rate of 0.2 s^−1^. (**b**) Effect of the strain rate on the tensile behavior of the fluorinated poly(ethyl acrylate-*random*-methyl acrylate) (PEMA) specimen PEMA4F21. Chemically crosslinked PEMA was stretched at 0.2 s^−1^. (**c**) In situ small-angle X-ray scattering (SAXS) measurement for PEMA4F21 under stretching at 0.1 and 0.01 s^−1^ in the horizontal direction. Time-resolved 2D-SAXS patterns were collected simultaneously with the stress–strain curve. 1D-SAXS profiles were extracted from the meridian and equatorial directions (± 10°) in the 2D-SAXS pattern and simulated using the Yarusso–Cooper model^51^. In the setup, the stretching direction was along the equatorial direction. The radius (*R*_1_), volume (*V*), and number density (*ND*) of the fluoroalkyl domains for each direction are plotted as a function of strain. The magenta and dark cyan plots correspond to the data stretched at 0.1 and 0.01 s^−1^, respectively. The structure of the domains under each stretching is schematically illustrated.

The network rearrangement behavior affects the mechanical properties of our fluorinated elastomers. As the network undergoes moderate rearrangement, not only the mechanical strength is enhanced but also several functions such as fracture resistance, high stretchability, and self-healing capability. In fact, PEMA4F21 exhibits high fracture stress (≈2 MPa) with high stretchability (≈1200%) due to the moderate network rearrangement at room temperature, whereas PEMA is purely viscous (Fig. 3a). The relaxation time τ (= ω^−1^) for network rearrangement at room temperature (28 °C) is 5.3 × 10^−1^ s in PEMA4F21, whereas that for the segmental motion of the polymer backbone is 2.3 × 10^−6^ s (Supplementary Fig. 9). PEA4F21 is viscous at room temperature because of its active network rearrangement. However, PMA4F21 exhibits a very high fracture stress (≈5 MPa) with short stretching (≈500%) due to slow network rearrangement at room temperature. The crystallization of fluoroalkyl side chains also restricts the network rearrangement; consequently, PEMA6F21, PEMA8F21, and PEMA14F21 exhibit higher moduli with lower stretchability (Supplementary Figs. 6 and 10).

PEMA4F21 sensitively changes its mechanical strength, modulus, and stretchability with strain rate at room temperature (Fig. 3b). When PEMA4F21 is stretched slowly, very high stretchability (more than 2300%) with low stress is achieved, whereas it behaves as an elastic material with high fracture stress (≈2 MPa) under fast deformation. Moreover, due to the dynamic nature of the crosslinks, PEMA4F21 is stronger and tougher than PEMA chemically crosslinked via covalent bonds (Fig. 3b). The chemical structure of chemically crosslinked PEMA is shown in Supplementary Fig. 1. The chemically crosslinked PEMA is stretched at a strain rate of 0.2 s^−1^ (Fig. 3b). PEMA4F21 exhibits 28 times higher toughness (≈17 MJ m^−3^) than chemically crosslinked PEMA. The toughening of elastomers by introducing detachable crosslinks is frequently observed^17,26,28,34–37,55–58^. In these cases, dissipation of locally concentrated stress upon detachment of physical crosslinks under deformation is expected. However, direct observation of the action of detachable crosslinks is challenging; instead, plausible mechanisms are generally provided.

In this work, we directly observed the detachment of highly stressed fluoroalkyl crosslinks in PEMA4F21 under stretching using in situ synchrotron SAXS. We collected in real time the two-dimensional (2D)-SAXS patterns of PEMA4F21 stretched to the horizontal direction within 1 s (Fig. 3c and Supplementary Fig. 11) and simulated the one-dimensional (1D)-SAXS profiles extracted from the meridian and equatorial directions in the 2D-SAXS pattern using the Yarusso–Cooper model^51^. In our experiment, the stretching direction was along the equatorial direction. Using this procedure, we obtained the *R*_1_, *R*_CA_, and *ND* values of the fluoroalkyl domains for each direction. In the analysis, we assumed that the domain was prolate spheroid-shaped to calculate its volume (*V*) under stretching. Thus, when PEMA4F21 is quickly stretched at a strain rate of 0.1 s^−1^, the fluoroalkyl domain is elongated along the stretching direction and the volume of the domain decreases. This indicates that highly stressed fluoroalkyl side chains detach from the domain as illustrated in Fig. 3c. At the same time, the *ND* in the direction normal to the stretching increases, which constitutes direct evidence for the detachment of highly stressed fluoroalkyl side chains from the domain and their resegregation into new domains under stretching. Owing to this phenomenon, the excess stress locally applied to the crosslinks in PEMA4F21 is dissipated, thereby preventing the fracture of the material, which contrasts with the case of chemically crosslinked PEMA (Fig. 3b). The dissipation of the high local stress endows the material with toughness, stretchability, and strength. Meanwhile, when PEMA4F21 is slowly stretched at a rate of 0.01 s^−1^, less stress is exhibited in the material because the external force applied is dissipated through the rearrangement of the network. In this case, the fluoroalkyl domain is somewhat elongated along the stretching direction, but the volume of the domain remains almost constant during the stretching. Despite the network rearrangement, the material maintains tension due to the persistent network structure; thus, very high stretchability with low stress is achieved.

### Self-healing properties

**Figure 4:**
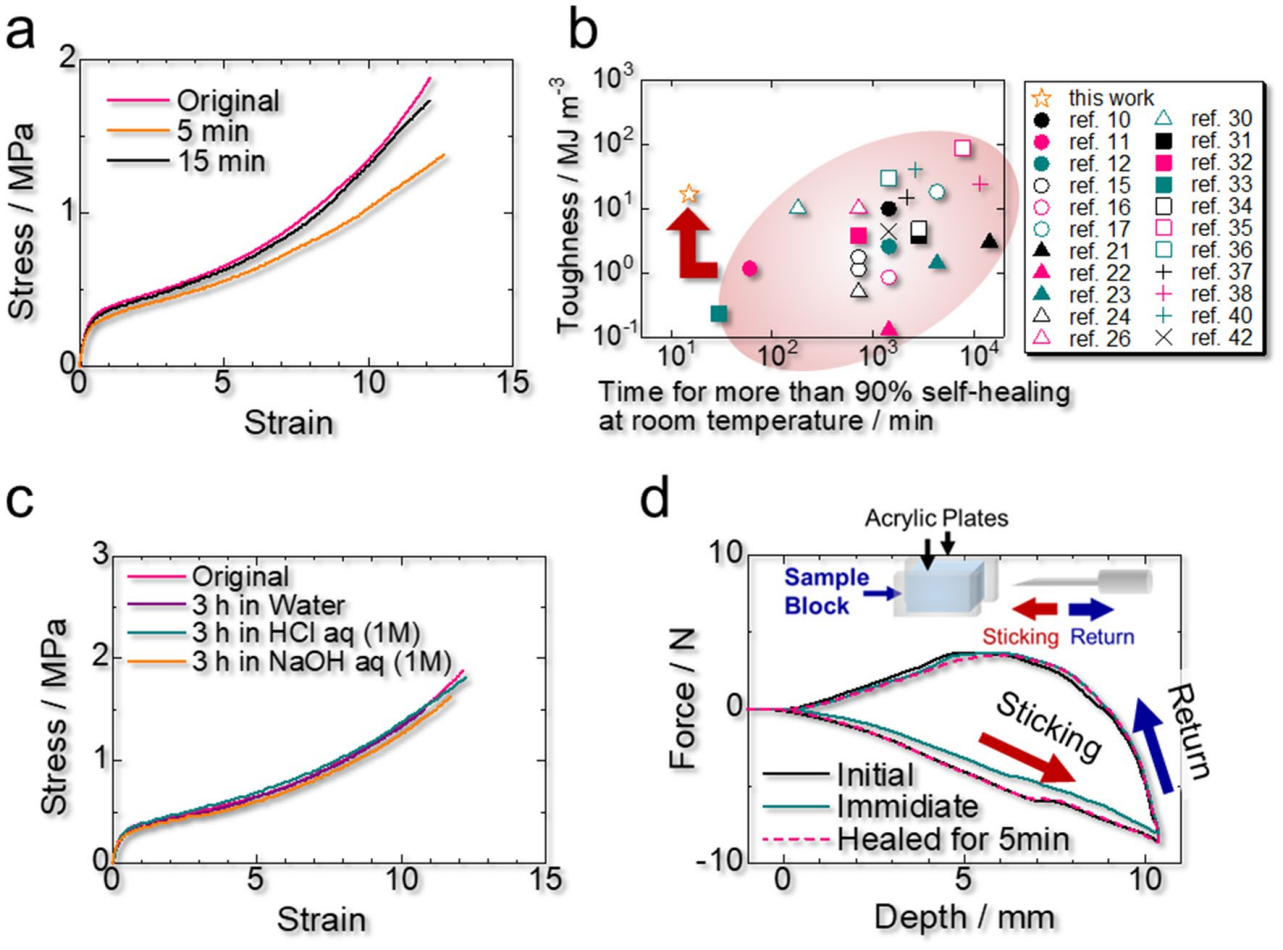
Self-healing behavior of PEMA4F21. (**a**) Stress–strain curves of cut fluorinated poly(ethyl acrylate-*random*-methyl acrylate) PEMA4F21 healed at 27 °C ± 1 °C for 5 and 15 min. (**b**) Ashby plot of toughness vs. time for more than 90% self-healing at room temperature for PEMA4F21 and other reported autonomously self-healable elastomers. (**c**) Self-healing behavior of PEMA4F21 immersed for 3 h in water and strongly acidic and basic aqueous solutions. (**d**) Self-healing behavior of PEMA4F21 damaged by needle sticking at 1 mm s^−1^. The damaged PEMA4F21 was allowed to self-heal at 27 °C ± 1 °C for 5 min.

The above results demonstrate that the trade-off relationship between the mechanical property and autonomous healing speed is circumvented for PEMA4F21. As shown in Fig. 1b and Supplementary Movie S1, when a PEMA4F21 sheet is cut using a razor, the cut faces reconnect with each other readily and strongly. At 27 °C ± 1 °C, the healing efficiencies of cut PEMA4F21 are 60% ± 20% and 91% ± 5% after 5 and 15 min, respectively (Fig. 4a). PEMA4F21 rapidly self-heals and recovers the original strength while maintaining good mechanical property. The Ashby plot of toughness vs. time required for more than 90% self-healing at room temperature depicted in Fig. 4b for PEMA4F21 and other reported autonomously self-healable elastomers confirms that this material overcomes the conventional trade-off relationship between the mechanical property and autonomous self-healing speed.

According to DFT calculations (Supplementary Fig. 4), the weak but abundant interchain van der Waals interactions between PEMA segments are the main driving force for the self-healing in PEMA4F21. A similar van der Waals self-healing mechanism has been reported for acrylic-based^45–48^ and polystyrene-*block*-polybutadiene-*block*-polystyrene-based^49,50^ polymers. PEMA4F21 exhibits not only better mechanical property but also faster self-healing than PEMA, whose healing efficiencies are 60% ± 20% and 90% ± 20% after 15 min and 1 h, respectively (Supplementary Fig. 12), which suggests that the fluoroalkyl crosslinks enhance both the mechanical strength and self-healing speed of the material. This contradictory effect is achieved by the autonomous rearrangement of the network between the damaged faces, which accelerates the approach between PEMA segments via the segmental diffusion of the polymer chains (Fig. 1c). If the network rearrangement is inactive at room temperature, the self-healing is poor. For example, the fluoroalkyl component of PEMA8F21 is crystalline and its healing efficiency is only 23% ± 9% after 17 h (Supplementary Fig. 13).

Since the self-healing of PEMA4F21 proceeds via interchain van der Waals interactions, the material self-heals even in water and strong acidic and basic aqueous solutions (Fig. 4c). Moreover, the needle sticking damage in PEMA4F21 is completely self-healed within only 5 min at room temperature (27 °C ± 1 °C) (Fig. 4d and Supplementary Movie S2), whereas a commercially available silicone elastomer does not heal under the same conditions (Supplementary Fig. 14). These results demonstrate the potential of this material for self-healing coating applications for electronic and medical equipment, *e.g.*, recyclable medical simulators in surgery.

### Advanced functions endowed by the fluoroalkyl components

**Figure 5:**
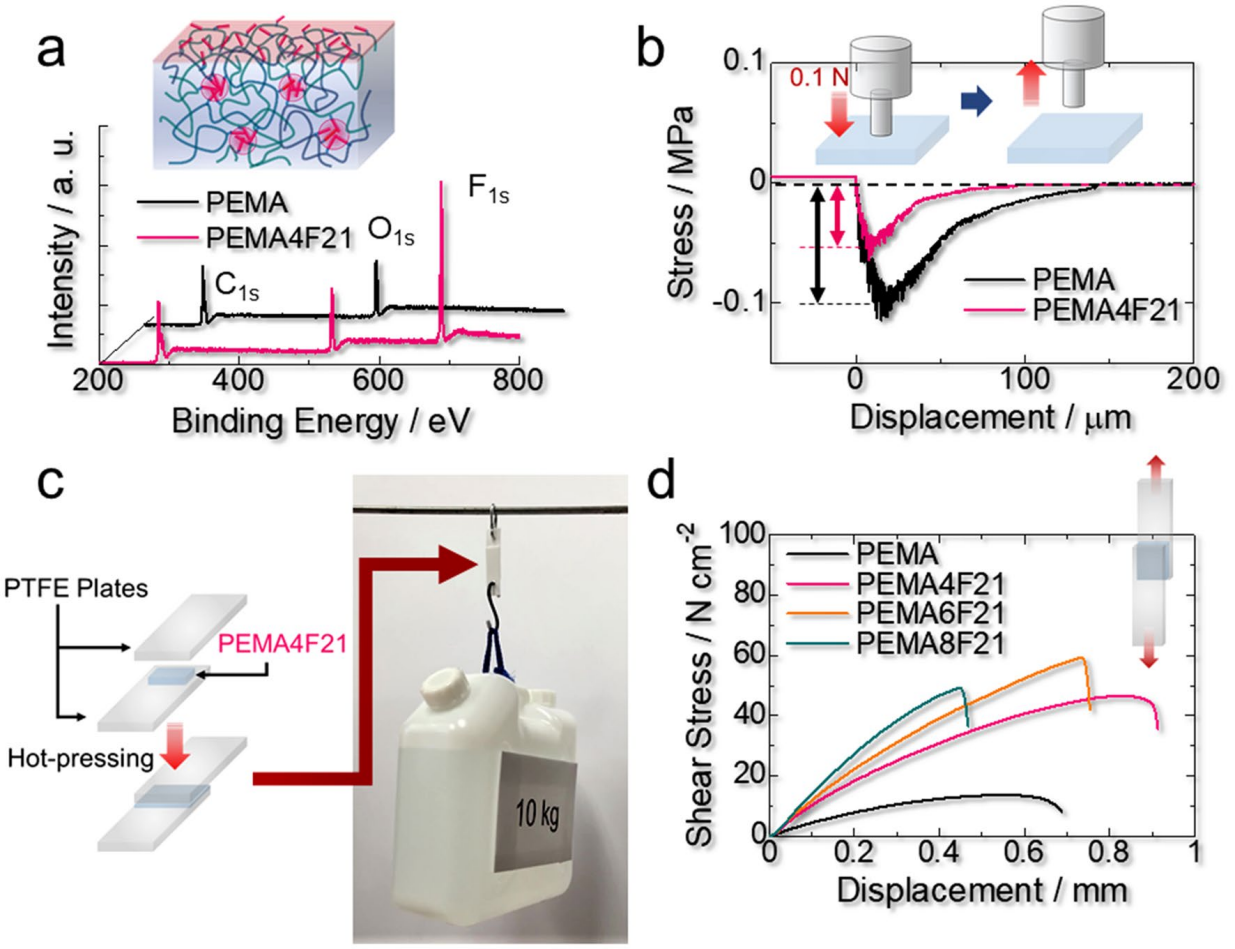
Advanced functions of PEMA4F21 based on the fluoroalkyl component. (**a**) X-ray photoelectron spectra demonstrating the surface segregation of the fluoroalkyl component in the fluorinated poly(ethyl acrylate-*random*-methyl acrylate) (PEMA) specimen PEMA4F21. (**b**) Adhesion of PEMA4F21 and PEMA sheets. A probe with a diameter of 4 mm was contacted to the sample surface at 0.1 N for 30 s and detached at 10 μm s^−1^. The arrow indicates the magnitude of adhesion. (**c**) A photograph of polytetrafluoroethylene (PTFE) plates adhered by PEMA4F21 lifting a 10 kg container. The adhesion area was 2 cm × 4 cm. (**d**) Shear stress for PTFE plates adhered by the indicated samples. The specimen was stretched at 10 mm min^−1^ and 27 °C ± 1 °C.

The intrinsic properties of the fluoroalkyls, such as nonstick and antiadhesive properties, chemical and thermal stabilities, and extremely low surface tension, endow the elastomer with unique functions. According to an X-ray photoelectron spectroscopy analysis, the fluorine atom abundance relative to carbons (F_1s_/C_1s_, where 1 s represents the atomic orbital) in PEMA4F21 is 1.7, whereas the assumed value for the bulk polymer is only 0.17 (Fig. 5a). Since the fluoroalkyl component is enriched at the surface of PEMA4F21, the adhesion of this polymer (0.05 MPa) is significantly lower than that of PEMA (0.1 MPa) (Fig. 5b). In contrast with other autonomous self-healing materials^31,36^, the surface of PEMA4F21 is selectively sticky only at the damaged faces (Supplementary Movie S1 and Supplementary Fig. 15). Considering the practical applications of autonomous self-healing materials, the stability of the surface is important to prevent undesired fusions. Meanwhile, the fluoroalkyl component enhances attractiveness to PTFE, which is known to have an extremely nonsticky and water-and-oil repellent surface. Our fluorinated polymers strongly adhere to PTFE plates via hot pressing (Fig. 5c, d, and Supplementary Movie S3). The adhesion strength under shear loading^59–61^ depends on the polymer composition, with PEMA6F21 exhibiting the maximum strength of *ca.* 60 N cm^−2^.

## Discussion

In this study, we developed a fluorinated elastomer that exhibits both ultrafast self-healing property and high mechanical strength on the basis of an “inversion” design contrary to conventional autonomous self-healing elastomers. The key aspect of the material design is the dynamic crosslinking of attractive PEMA chains via repulsive segregation of fluoroalkyl side chains. In this material, the self-healing proceeds via weak but abundant van der Waals interactions between PEMA segments in the matrix, while the crosslinking ensures the strength of the material. Furthermore, the fluoroalkyl component covers and protects the material surface and provides it with selective self-healing ability between the damaged faces. Importantly, this simple material design can be expected to endow other adhesive polymers with elastic and self-healing capabilities. Further studies are currently underway in our laboratory and will be reported in due course.

## Methods

Details of sample synthesis, structural characterizations, and measurements are described in the Supplementary Information.

### Materials

Inhibitors were removed from methyl acrylate (MA, > 99.0%, Tokyo Chemical Industry), ethyl acrylate (EA, > 99%, Nakalai Tesque), 1H,1H,2H,2H-perfluorododecyl acrylate (C_10_F_21_A, 97%, Apollo Scientific), 1H,1H,2H,2H-heptadecafluorodecyl acrylate (C_8_F_17_A, > 97.0%, Tokyo Chemical Industry), 2-(perfluoro-9-methyldecyl)ethyl acrylate (C_11_F_23_A, 97%, Apollo Scientific), and 1,9-bis(acryloyloxy)nonane (> 92.0%, Tokyo Chemical Industry) using aluminum oxide (activated, Kanto Chemical) before use. Benzoyl peroxide (BPO, containing 25 wt% water, Nakalai Tesque) was purified via recrystallization using Drysol N (ethanol 88%, methanol 3%, 2-propanol 9%, Kanto Chemical). Toluene (> 99.5%, Kanto Chemical), *n*-hexane (> 96.0%, Kanto Chemical), tetrahydrofuran (THF, > 99.5%, Kanto Chemical), acetone (> 99.5%, Kanto Chemical), methanol (> 99.8%, Kanto Chemical), hydrochloric acid (35%, Nacalai Tesque), CDCl_3_ (99.8%, Nacalai Tesque), sodium hydroxide (NaOH, 97%, Nakalai Tesque), and 2-benzyl-2-(dimethylamino)-4'-morpholinobutyrophenone (> 98.0%, Tokyo Chemical Industry) were used without further purification. A chemically crosslinked silicone elastomer block was obtained from Tigers Polymer Co.

### Preparation of polymers

All polymers used in this work were prepared via radical polymerization under N_2_ atmosphere using benzyl peroxide as an initiator. The molecular weights and the polydispersity indices of the polymers were determined by GPC calibrated with polystyrene standards. The compositions of the polymers were determined by ^1^H NMR spectroscopy.

### Morphology characterization

SAXS measurements were performed at the BL-15A2 beamline at the Photon Factory of the High Energy Accelerator Research Organization in Tsukuba, Japan. The wavelength (*λ*) of the synchrotron radiation X-ray was 0.12 nm. PILATUS 2 M was used as a detector and calibrated using stearic acid and silver behenate as standards. In the static SAXS measurement, the 1D-SAXS pattern with scattering vector *q* (*q* = (4π/*λ*) sinθ where 2θ is the scattering angle) was obtained via circular averaging of the experimentally obtained 2D-SAXS pattern. The background scattering was subtracted from the sample scattering taking the sample absorption into consideration.

### Viscoelastic measurement

DMA measurements were conducted on a DMA Q800 manufactured by TA Instruments. A tensile strain of 0.1% was applied to a rectangular-shaped sample sheet at 1 Hz. The sample sheet was heated from − 50 to 100 °C at a heating rate of 2 °C min^−1^.

### Mechanical test

The tensile stress–strain curves of the sample sheets were collected at 27 °C ± 1 °C using a force tester MCT-2150 (A&D Co., Ltd.) and a tensile-compression testing apparatus manufactured by AcroEdge Co., Ltd. The test pieces were cut into JIS 7-based dumbbell-shapes with waist dimensions of 5.0 mm × 2.0 mm × 0.5 mm. The test pieces were typically stretched at a strain rate of 0.2 s^−1^. Measurements were performed at least trice under the same condition. The stress was calculated as *F*/*S*_0_, where *F* is the loading force, and *S*_0_ is the initial cross-sectional area of the sample piece. The strain was calculated from the marker distance (*l*) after stretching, relative to the initial marker distance (*l*_0_) of the specimen, i.e., (*l* − *l*_0_) *l*_0_^−1^. *l* and *l*_0_ were recorded using a digital camera.

### In situ SAXS measurement under stretching

In situ SAXS measurements were conducted on an AcroEdge tensile-compression testing apparatus installed onto the BL-15A2 beamline. Dumbbell-shaped PEMA4F21 sheets were stretched at strain rates of 0.1 and 0.01 s^−1^ in the horizontal direction. 2D-SAXS patterns were accumulated for every exposure time of 1 s during the stretching. The 2D-SAXS patterns obtained simultaneously with the stress–strain curve were sector-averaged for meridian and equatorial directions within ± 10° to obtain 1D-SAXS profiles. In this analysis, the equatorial direction corresponds to the stretching direction.

### Self-healing tests after cutting

The self-healing of a cut sample sheet was estimated via tensile testing at 27 °C ± 1 °C. Typically, a sample sheet was cut, leaving a thickness of 12.5 μm, using a razor and a spacer to prevent the sheet from being completely separated into two pieces. The cut faces were gently contacted with each other. To remove noncontact spots between the contact faces, the sample piece was evaporated for 1 min in a glass desiccator. Then, the sample piece was stored at room temperature (27 °C ± 1 °C) in air for different periods of time. The healed sample sheets were then stretched at a strain rate of 0.2 s^−1^. The self-healing efficiency was defined as the percentage of the tension energy required to break the self-healed sheet against that of the original sheet. The tension energies were measured as the area below the stress–strain curve.

### Self-healing tests in water and HCl and NaOH aqueous solutions

PEMA4F21 sheets were immersed in water and HCl (1 M) and NaOH (1 M) aqueous solutions in a Petri dish. The sample sheets were cut using a razor and the cut faces were gently contacted in the solutions. The cut sheets were allowed to self-heal at room temperature (27 °C ± 1 °C) for 3 h in the solutions. Then, the healed sheets were vacuum dried for 5 min after rinsing with water. The healed sample sheets were then stretched at 0.2 s^−1^.

### Self-healing tests after sticking

The self-healing tests of PEMA4F21 and commercially available chemically crosslinked silicone elastomer (Tiger Polymers Co.) blocks damaged by a sticking needle were conducted on an AcroEdge tensile- compression tester. A sample block held between transparent acrylic plates was stuck by an injection needle (0.6 mm ϕ) to a depth of 10 mm approximately at 1 mm s^−1^. The needle was immediately pulled out from the specimen at the same speed. This cycle was immediately repeated. Subsequently, the sample was allowed to heal at 27 °C ± 1 °C for 5 and 15 min, and the needle was then stuck again at the same position.

### Probe tack test

The probe tack test was performed using an Anton Paar MCR302 rheometer. A sample sheet with a thickness of 0.5 mm deposited on a glass slide was fixed on a Peltier cooler stage kept at 25 °C. A parallel plate with a diameter of 4 mm was contacted to the sample surface at 0.1 N for 30 s and detached at a rate of 10 μm s^−1^. The load during the action was recorded.

### Adhesive test

A sample polymer sheet placed between PTFE plates was pressed at 100 °C. The polymer layer was ~ 250 mm thick. The adhesion area was fixed as 10 mm × 10 mm. The specimen was stretched at 10 mm min^−1^ using the MCT-2150 tester, and the tensile stress–strain curves were collected.

## Supplementary Information

Supplementary Video 1.

Supplementary Video 2.

Supplementary Video 3.

Supplementary Information 1.

## Data Availability

The authors declare that the data supporting the findings of this study are available within the article and its Supplementary Information files or are available from the authors upon reasonable request.
