# Supplementary material for: Repulsive segregation of fluoroalkyl side chains turns a cohesive polymer into a mechanically tough, ultrafast self-healable, nonsticky elastomer
Source: Sci Rep. 2022 Jul 25;12:12009. doi: 10.1038/s41598-022-16156-9 (PMC9314360; doi:10.1038/s41598-022-16156-9)
Supplement: Supplementary file 4 — Supplementary Information 1. [file 41598_2022_16156_MOESM4_ESM.pdf]

Supplementary Information for

Repulsive segregation of fluoroalkyl side chains turns a cohesive polymer into a mechanically tough, ultrafast self-healable, nonsticky elastomer

Yohei Miwa[1,†,], Taro Udagawa[1], and Shoichi Kutsumizu[1]*

[1]Department of Chemistry and Biomolecular Science, Faculty of Engineering, Gifu University, Yanagido, Gifu 501-1193, Japan.

[†]PRESTO, Japan Science and Technology Agency.

*Author to whom correspondence should be addressed.
E-mail: y_miwa@gifu-u.ac.jp

Table of Contents

1. Supplementary methods

   1. 1 Materials synthesis

   1. 2 Measurements

   1. 3 Computational details

   1. 4 Self-healing tests

2. Supplementary table and figures

3. References

1. Supplementary methods

1. 1 Materials synthesis

Preparation of polymers. The synthesis route of the fluorinated poly(ethyl acrylate-*random*-methyl acrylate) (PEMA) polymer PEMA*x*F*y* is shown in Supplementary Fig. 1. All polyacrylates used in this work were typically synthesized, purified, and dried as follows: A reaction mixture containing the appropriate monomers was transferred to a three-neck flask. The flask was sealed after bubbling with N_2 for more than 30 min. The mixture was heated at 60°C for more than 24 h in N_2 atmosphere using a water bath. After the polymerization, the reaction mixture was dissolved in acetone and the solution was poured into a large amount of stirred *n*-hexane. The obtained precipitate was dried at 100°C for more than 24 h under vacuum.

Polymerization of PEMA. Inhibitors were removed from a mixture of MA (13.9 g, 162 mmol), EA (16.2 g, 162 mmol), and toluene (15 mL) using aluminum oxide in a vial. Then, purified BPO (78.1 mg, 0.322 mmol) was dissolved in the mixture. After filtration, the mixture was transferred to a three-neck flask and polymerized, purified, and dried following the procedure described above. The weight average molecular weight (M_w) and the polydispersity index (M_w/M_n) determined by gel permeation chromatography (GPC) were 425,200 and 6.72, respectively. The actual concentration of MA unit determined by 1H nuclear magnetic resonance (NMR) was 45 mol%. ^{1}H NMR (400 MHz, $CDCl_3$, ppm) (Supplementary Fig. 2B): δ 1.25 (methyl protons of the EA unit, 3H), 1.35−2.00 (methylene protons of the copolymer backbone, 2H), 2.30 (methine proton of the backbone, 1H), 3.64 (oxymethyl protons of the MA unit, 3H), and 4.11 (oxymethylene protons of the EA unit, 2H).

Polymerization of PEMA*x*F*y*. For the polymerization of PEMA*x*F*y*, the [MA]/[EA] and [monomer]/[BPO] ratios were fixed at 1 and 1000, respectively. $C_8F_{17}A$, $C_{10}F_{21}A$, and $C_{11}F_{23}A$ were used as monomers to prepare PEMA*x*F17, PEMA*x*F21, and PEMA*x*F23, respectively. Taking PEMA4F21 as an example, the polymerization was conducted as follows: Inhibitors were removed from a mixture of MA (9.61 g, 112 mmol), EA (11.2 g, 112 mmol), $C_{10}F_{21}A$ (5.75g, 9.30 mol), and toluene (20 mL) using aluminum oxide in a vial. Then, purified BPO (56.3 mg, 0.232 mmol) was dissolved in the mixture. After filtration, the mixture was transferred to a three-neck flask and subjected to the polymerization reaction followed by purification and drying according to the procedure described above. The M_w and M_w/M_n were determined by GPC to be 379,000 and 4.11,

respectively. The actual concentrations of MA, EA, and $C_{10}F_{21}A$ units determined by ^1H NMR were 45, 51, and 4 mol%, respectively. ^1H NMR (400 MHz, $CDCl_3$, ppm) (Supplementary Fig. 2A): δ 1.25 (methyl protons of the EA unit, 3H), 1.35−2.00 (methylene protons of the copolymer backbone, 2H), 2.30 (methine proton of the backbone, 1H), 2.51 (methylene protons of the $C_{10}F_{21}A$ unit, 2H), 3.65 (oxymethyl protons of the MA unit, 3H), 4.11 (oxymethylene protons of the EA unit, 2H), and 4.35 (oxymethylene protons of the $C_{10}F_{21}A$ unit, 2H). The asymmetric (ν_aCF_2) and symmetric CF_2 stretching vibration (ν_sCF_2) bands were observed at 1208 and 1152 cm^{-1}, respectively, in the Fourier-transform infrared (FT-IR) spectrum[1] (Supplementary Fig. 3A).

Polymerization of PF21. $C_{10}F_{21}A$ (1.96 g, 3.17 mmol) dissolved in toluene (2 mL) was purified using aluminum oxide to remove the inhibitor. Then, purified BPO (0.9 mg, 4 μmol) was dissolved in the solution. After filtration, the solution was transferred to a three-neck flask. The reaction mixture in the flask was polymerized, purified, and dried following the procedure described above. The insolubility of the polymer in THF and chloroform prevented its characterization via GPC and ^1H NMR measurements. The FT-IR spectrum exhibited ν_aCF_2, ν_sCF_2, and symmetric CF_3 vibration (ν_sCF_3) bands at 1208, 1152, and 1344 cm^{-1}, respectively (Supplementary Fig. 3A).

Polymerization of PMA. Inhibitors were removed from a mixture of MA (14.9 g, 173 mmol) and toluene (15 mL) using aluminum oxide in a vial. Then, purified BPO (42.2 mg, 0.174 mmol) was dissolved in the mixture. After filtration, the mixture was transferred to a three-neck flask and polymerized, purified, and dried following the procedure described above. The M_w and M_w/M_n determined by GPC were 235,700 and 3.51, respectively. ^1H NMR (400 MHz, $CDCl_3$, ppm) (Supplementary Fig. 2B): δ 1.35−2.00 (methylene protons of the backbone, 2H), 2.30 (methine proton of the backbone, 1H), and 3.64 (oxymethyl protons, 3H).

Polymerization of PMA4F21. Inhibitors were removed from a mixture of MA (9.45 g, 110 mmol), $C_{10}F_{21}A$ (2.80 g, 4.53 mmol), and toluene (10 mL) using aluminum oxide in a vial, and purified BPO (27.4 mg, 0.113 mmol) was dissolved in the mixture. The mixture was filtrated and transferred to a three-neck flask, where it was polymerized, purified, and dried following the procedure described above. The M_w and M_w/M_n determined by GPC were 274,000 and 4.20, respectively. ^1H NMR (400 MHz, $CDCl_3$, ppm) (Supplementary Fig. 2B): δ 1.35−2.00 (methylene protons of the copolymer backbone, 2H), 2.30 (methine proton of the backbone, 1H), 2.51 (methylene protons of

the $C_{10}F_{21}A$ unit, 2H), 3.65 (oxymethyl protons of the MA unit, 3H), and 4.35 (oxymethylene protons of the $C_{10}F_{21}A$ unit, 2H). The ν_aCF_2 band was detected at 1208 cm^{-1} in the FT-IR spectrum (Supplementary Fig. 3B).

Polymerization of PEA. Inhibitors were removed from a mixture of EA (20.1 g, 201 mmol) and toluene (20 mL) using aluminum oxide in a vial. Then, purified BPO (24.0 mg, 99.1 μmol) was dissolved in the mixture. After filtration, the mixture was transferred to a three-neck flask, and polymerized, purified, and dried following the procedure described above. The M_w and M_w/M_n were determined by GPC to be 358,800 and 4.53, respectively. ^{1}H NMR (400 MHz, $CDCl_3$, ppm) (Supplementary Fig. 2B): δ 1.25 (methyl protons, 3H), 1.35−2.00 (methylene protons of the backbone, 2H), 2.30 (methine proton of the backbone, 1H), and 4.11 (oxymethylene protons, 2H).

Polymerization of PEA4F21. A mixture of EA (7.88 g, 78.7 mmol), $C_{10}F_{21}A$ (2.01 g, 3.26 mmol), and toluene (10 mL) was treated with aluminum oxide in a vial to remove the inhibitors. Then, purified BPO (1.8 mg, 7.4 μmol) was added to the mixture. After filtration, the mixture was transferred to a three-neck flask to conduct the polymerization, purification, and drying as described above. The M_w and M_w/M_n determined by GPC were 248,900 and 3.97, respectively. ^{1}H NMR (400 MHz, $CDCl_3$, ppm) (Supplementary Fig. 2B): δ 1.25 (methyl protons of the EA unit, 3H), 1.35−2.00 (methylene protons of the copolymer backbone, 2H), 2.30 (methine proton of the backbone, 1H), 2.51 (methylene protons of the $C_{10}F_{21}A$ unit, 2H), 4.11 (oxymethylene protons of the EA unit, 2H), and 4.35 (oxymethylene protons of the $C_{10}F_{21}A$ unit, 2H). The ν_aCF_2 band was detected at 1208 cm^{-1} in the FT-IR spectrum (Supplementary Fig. 3C).

Photopolymerization of chemically crosslinked PEMA. A mixture of MA (1.39 g, 16.2 mmol), EA (1.61 g, 16.1 mmol), and 1,9-bis(acryloyloxy)nonane (0.178 g, 0.664 mmol) was treated with aluminum oxide in a vial to remove the inhibitors. Then, 2-benzyl-2-(dimethylamino)-4'-morpholinobutyrophenone (11.7 mg, 31.9 μmol) was dissolved in the mixture. The mixture was bubbled with Ar for more than 30 min. After filtration, the mixture was transferred to a Teflon Petri dish and photopolymerized under UV irradiation using an ultra-high-pressure mercury lamp (Ushio, SX-UI501HQ, 500 W) in Ar atmosphere. The obtained polymer sheet was dried under vacuum at 100°C for 24 h.

Preparation of the sample sheets. The polymers were dissolved in THF at a concentration of 10 wt%. The solution was poured into a Teflon Petri dish and slowly

dried at room temperature to prepare a cast sheet, which was further dried at 100°C for more than 24 h and then at 35°C for 24 h under vacuum. The sheet thickness was approximately 0.5 mm.

Preparation of the sample blocks. A block of PEMA4F21 with a thickness of 5 mm was prepared by heat press molding at 100°C.

1. 2 Measurements

^{1}H NMR spectroscopy. The ^{1}H NMR spectra were obtained on a JEOL ECX-400P (400 MHz) spectrometer. The samples were dissolved in $CDCl_3$ containing tetramethylsilane as an internal standard.

GPC measurements. The GPC measurements were performed using an EXTREMA HPLC system manufactured by JASCO, equipped with a polystyrene gel column (Shodex GPC LF-804). THF was used as the eluent at 40°C. The column set was calibrated using standard polystyrene (Tosoh) samples with small polydispersity indices.

Differential scanning calorimetry (DSC). A DSC7020 instrument manufactured by SII was calibrated with indium, zinc, lead, and tin standards. A sample contained in an aluminum pan was heated from −80°C to 170°C at a rate of 10°C min^{-1}. The temperature corresponding to half the endothermic shift was defined as the glass transition temperature (T_g). During the measurements, dry N_2 gas was flowed at a rate of 40 mL min^{-1}.

FT-IR spectroscopy. The FT-IR spectra were obtained using a JASCO FT/IR6600 spectrometer equipped with a triglycine sulfate detector. A thin cast film was prepared on a KBr plate from a chloroform solution. The film was dried at 35°C overnight under vacuum. Measurements were performed in transmittance mode at a resolution of 4 cm^{-1} using 32 scans.

X-ray diffraction (XRD). XRD measurements were conducted on a RIGAKU RINT-2100 diffractometer with a CuKα X-ray (wavelength $\lambda = 0.154$ nm). The scattering angle (2θ) was scanned from 2° to 50° at a rate of 2.00° min^{-1}.

X-ray photoelectron spectroscopy (XPS). XPS measurements were conducted on a Quantera SXM-GS (ULVAC-PHI, Inc.) with an AlKα X-ray source operated at 15 kV

and 2 mA. The takeoff angle was kept constant at 45°. A sample thin film supported on a glass slide was prepared via spin coating from a THF solution. The spin coated film was dried at 60°C for 24 h under vacuum.

Small-angle X-ray scattering (SAXS). SAXS measurements were conducted on a BL-15A2 beamline at the Photon Factory of the High Energy Accelerator Research Organization in Tsukuba, Japan. The X-ray wavelength (λ) was 0.12 nm. A PILATUS-2M was used as a detector. The distance between the sample and the detector was approximately 1 m. Stearic acid and silver behenate were used as calibration standards for the detector. The background scattering and dark current data were subtracted from the sample data considering the sample absorption. Samples were irradiated with synchrotron X-ray for 10 s. The obtained two-dimensional (2D) SAXS pattern was radially integrated to make a circular average and converted to a one-dimensional (1D) SAXS pattern. The resulting 1D SAXS patterns were simulated using the Yarusso–Cooper model.[2] This model assumes that spheres with a radius R_1 randomly disperse with the closest approach limitation ($2R_{CA}$) in the polymer matrix. That is, a restricted polymer shell with a thickness of R_{CA}–R_1 is considered around the fluoroalkyl domain. The scattering intensity for this model can be expressed by the following equations:

$$I(q) = \frac{K}{V_p} V_1^2 \Phi(qR_1)^2 \frac{1}{1+(\frac{8V_{CA}}{V_p})\Phi(2qR_{CA})} \quad (1)$$

$$V_{CA} = \frac{4}{3}\pi R_{CA}^3 \quad (2)$$

$$V_1 = \frac{4}{3}\pi R_1^3 \quad (3)$$

$$\Phi(x) = 3\frac{\sin x - x\cos x}{x^3} \quad (4)$$

where V_p and K are the average sample volume occupied by one particle and an adjustable parameter for the intensity scale, respectively. The scattering vector q is defined as $(4\pi/\lambda)\sin\theta$, where 2θ is the scattering angle.

Dynamic mechanical analysis (DMA) measurements. DMA measurements were conducted on a DMA Q800 manufactured by TA Instruments. A tensile strain of 0.1%, within the linear viscoelasticity regime, was applied to a rectangular-shaped sample sheet at 1 Hz. In the temperature sweep test, the sample sheet was heated from −50°C to 100°C

at a heating rate of 2°C min^{-1}. The sample temperature was controlled by flowing N_2 gas generated from liquid N_2.

Tensile test. The tensile stress–strain curves of the sample films were collected using a force tester MCT-2150 (A&D Co., Ltd.) and a tensile-compression testing apparatus (AcroEdge Co., Ltd.) at 27°C ± 1°C. The test pieces were cut into JIS 7-based dumbbell-shape with the waist dimensions of 5.0 mm × 2.0 mm × 0.5 mm. The initial clamp distance was typically 11 mm. Measurements were performed at least three times at the same condition. The stress was calculated as F/S_0, where F is the loading force, and S_0 is the initial cross-sectional area of the sample piece. The strain was calculated from the marker distance (l) with stretching, relative to the initial marker distance (l_0) of the specimen, i.e., $(l - l_0)\ l_0^{-1}$. The l and l_0 were recorded using a digital camera.

Adhesive test. A sample polymer sheet placed between polytetrafluoroethylene (PTFE) plates with a thickness of 2 mm was pressed at 100°C using a spacer (4.25 mm). That is, the thickness of the polymer layer was ca. 250 μm. The adhesion area was fixed as 10 mm × 10 mm. The specimen was stretched at 10 mm min^{-1} using MCT-2150, and the corresponding tensile stress–strain curves were collected.

Rheological measurements. The rheological properties of the polymers were investigated using an Anton Paar MCR302 rheometer. A parallel plate with a diameter of 8 mm was used for the measurements. The gap was approximately 0.4 mm, and a strain of 0.1% was applied, which was within the linear viscoelasticity regime. Frequency sweep tests were conducted within a dynamic range from 0.01 to 50 Hz. The data were collected from −15°C to 100°C with an interval of 2.5°C.

Probe tack test. The probe tack test was performed using an Anton Paar MCR302 rheometer. A sample sheet with a thickness of 0.5 mm deposited on a glass slide was fixed on a Peltier cooler stage kept at 25°C using a homemade holder. A parallel plate with a diameter of 4 mm was used as a probe for the measurements. The probe was contacted to the sample surface at 0.1 N for 30 s as a preload before the detachment action. The detachment was performed at a rate of 10 μm s^{-1} and the load during the action was recorded.

***In situ* SAXS measurements.** *In situ* SAXS measurements were conducted on a tensile-compression testing apparatus (AcroEdge Co., Ltd.) installed onto the BL-15A2 beamline.

JIS 8-based dumbbell-shaped PEMA4F21 sheets with the waist dimensions of 10 mm × 4.0 mm × 0.5 mm were stretched at 25°C ± 1°C at strain rates of 0.1 and 0.01 s^{-1} in the horizontal direction. 2D SAXS patterns were accumulated every exposure time of 1 s during the stress−strain curve measurement. The X-ray wavelength was 0.12 nm. The distance between the sample and the detector (PILATUS-2M) was ~1 m. 2D SAXS patterns simultaneously obtained with the stress−strain curve were sector-averaged for meridian and equatorial directions within ±10° to obtain 1D SAXS profiles. In this analysis, the equatorial direction corresponds to the stretching direction. The 1D SAXS patterns were simulated according to the Yarusso–Cooper model.[2]

1. 3 Computational details

Density functional theory (DFT) calculations. To estimate the strength of the interaction between fluoroalkyl side chains and between PEMA units, DFT calculations were conducted. To reduce the computational cost and to simplify the discussion, a partially substituted methyl acrylate trimer, in which the second methyl side chain was replaced with a fluoroalkyl side chain (fluoroalkyl ester compound) or an ethyl side chain (alkyl ester compound), was used for the calculation.

The initial structures for each DFT calculation were prepared as follows. For the fluoroalkyl ester pair, a geometric optimization was first performed on the fluoroalkyl ester compound having a straight fluoroalkyl side chain. Then, the fluoroalkyl pair in which the optimized molecules are aligned and contact through the fluoroalkyl chains was adopted as the initial structure for the DFT calculation. The procedure for constructing the initial structure for the DFT calculation of the alkyl ester pair was more complicated due to the shortness of the ethyl side chain. First, a geometry optimization was performed for an ethane pair. Then, the alkyl ester pair structure was built with the aid of GaussView6 visualization software with fixing the coordinates of the ethane parts. To obtain the low-energy conformation of the alkyl ester pair, the anharmonic downward distortion following (ADDF)[3] calculation was performed using the global reaction route mapping (GRRM)[4] program. The ADDF calculation was conducted with LADD = 3, NLowest = 10, and EQOnly keywords to efficiently find the low-energy conformers. The BondNoRearrange keyword was also used to prevent covalent bonds in the given initial geometry from breaking, and the coordinates of the ethane parts were fixed during the ADDF calculation. The ADDF calculation was performed with the PM6 semiempirical molecular orbital method to give 10 equilibrium structures, and the lowest-energy conformer was used as the initial structure for the DFT calculation of the alkyl ester pair.

The ωB97XD exchange-correlation functional and the 6-31G** basis set were adopted for the DFT calculation. The counterpoise correction[5] was used to correct the basis set superposition error. All DFT and PM6 calculations were performed with the Gaussian09 program package. To visualize the intermolecular interactions, a noncovalent interaction (NCI) analysis[6-8] was performed for the DFT-optimized fluoroalkyl ester pair and alkyl ester pair compounds. In the 3D-NCI plot, blue, green, and red regions represent strong attractive interactions, weak interactions, and strong repulsive interactions, respectively. The interaction energy for the DFT-optimized alkyl ester pair compounds was calculated to be 45 kJ mol^{-1}. In the case of the fluoroalkyl ester pair, the interaction energy was selectively calculated only between the fluoroalkyl side chains in the optimized pair structure as follows: As a first step, the main-chain parts were removed from the DFT-optimized fluoroalkyl ester pair and only the fluoroalkyl side chains having a methyl group at the terminal were left. The interaction energy for the left fluoroalkyl side chains was calculated to be 23 kJ mol^{-1}.

1. 4 Self-healing tests

Self-healing tests after cutting. The self-healing behavior of a cut sheet was estimated via tensile testing at 27°C ± 1°C. Briefly, a sample sheet was cut leaving a thickness of 12.5 μm using a razor and a spacer to prevent the sheet from being completely separated into two pieces. The cut faces were gently contacted with each other. To remove noncontact spots between the contact faces, the sample piece was evaporated for 1 min in a glass desiccator. Then, the sample piece was stored at room temperature (27°C ± 1°C) in air for different periods of time. The healed sample pieces were then stretched at a strain rate of 0.2 s^{-1}. The self-healing efficiency was defined as the percentage of the tension energy required to break the self-healed sheet against that of the original noncut sheet. The tension energies were measured as the area below the stress–strain curve.

Self-healing tests in water and HCl and NaOH aqueous solutions. PEMA4F21 sheets were immersed in water and HCl (1 M) and NaOH (1 M) aqueous solutions in a Petri dish. The sample sheets were cut using a razor, and the cut faces were gently contacted in the solutions. The cut sheets were self-healed at room temperature (27°C ± 1°C) for 3 h in the solutions. Then, the healed sheets were vacuum-dried for 5 min after rinsing with water and wiping with a paper. The healed sample sheets were then stretched at a strain rate of 0.2 s^{-1}.

Self-healing tests between original surfaces. PEMA4F21 sheets were completely cut using a razor, and the original surfaces of the cut pieces were contacted with an area of 2.0 × 1.6 mm. The stacked sheets were stored at room temperature (27°C ± 1°C) for 1 h. Then, the sheets were stretched at a strain rate of 0.2 s^{-1}.

Self-healing tests after sticking. The self-healing tests of PEMA4F21 and commercially available chemically crosslinked silicone elastomer (Tiger Polymers Co.) blocks damaged by sticking a needle were performed on a tensile-compression tester manufacture by AcroEdge. The sample blocks were hold between clear acrylic plates and stuck by an injection needle (0.6 mm ϕ) to a depth of 10 mm approximately at 1 mm s^{-1}. The needle was immediately pulled out from the sample at the same speed. This cycle was immediately repeated. Then, the PEMA4F21 and silicone blocks were healed at 27°C ± 1°C for 5 and 15 min, respectively. After the healing, the needle was stuck in sample blocks at the same position.

2. **Supplementary table and figures**

Supplementary Table 1. Summary of molecular characteristics of the prepared

Notation	M_w[a]	M_w/M_n[a]	Fluoroalkyl unit[b] / mol%	MA unit[b] / mol%	EA unit[b] / mol%	Fluoroalkyl unit[b] / wt%
PEMA6F21	247,200	5.14	6	43	51	30
PEMA8F21	295,700	3.52	8	42	50	37
PEMA14F21	140,700	3.03	14	36	50	52

[a]Measured by GPC using polystyrene standards. [b]Determined by ^{1}H NMR.

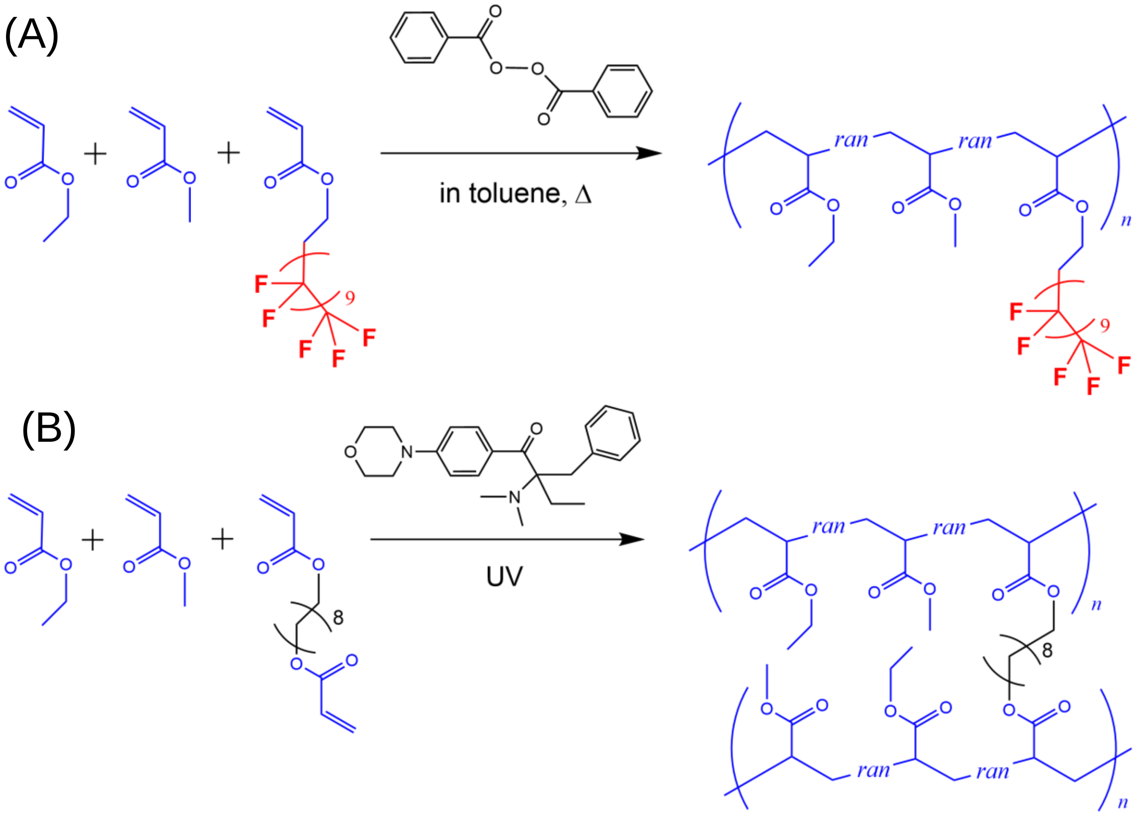

Supplementary Fig. 1. Synthetic route of (A) fluorinated poly(ethyl acrylate-*random*-methyl acrylate) (PEMA) polymers PEMA*x*F*y* and (B) chemically crosslinked PEMA.

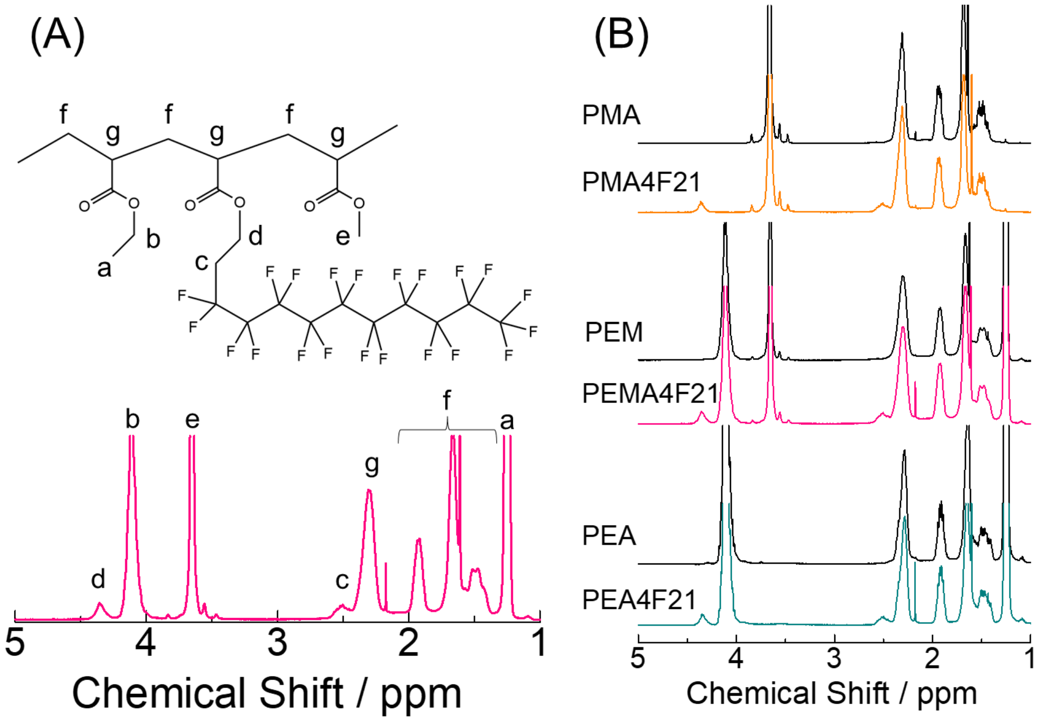

Supplementary Fig. 2. (A) Assignment of the ^{1}H nuclear magnetic resonance (NMR) peaks of PEMA4F21. (B) ^{1}H NMR of the indicated samples.

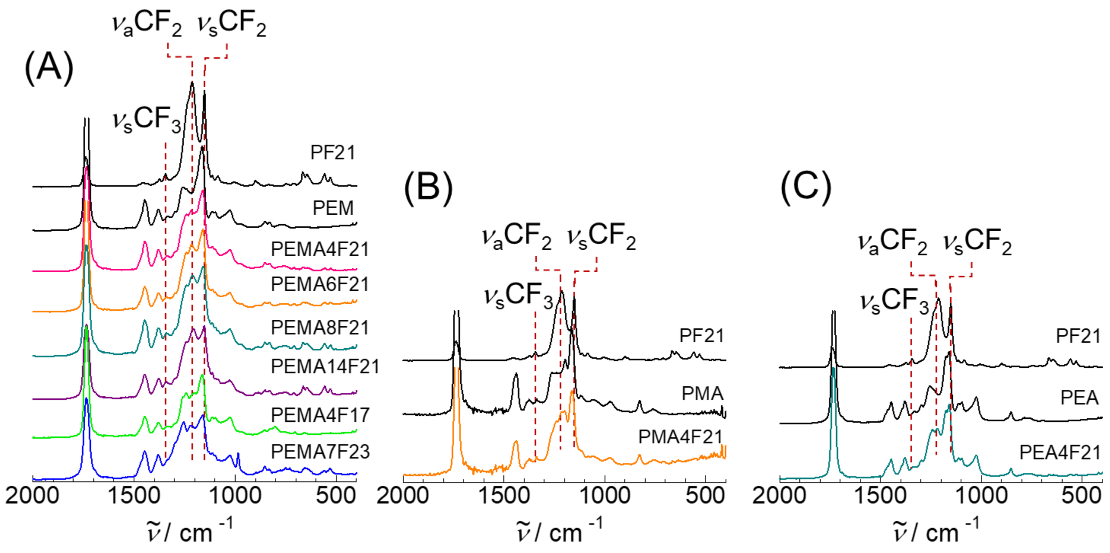

Supplementary Fig. 3. Fourier transform infrared spectra of the (A) PEMA*x*F*y*, (B) PMA4F21, and (C) PEA4F21. The stretching vibration bands in the fluoroalkyl side chains are indicated with dashed lines.

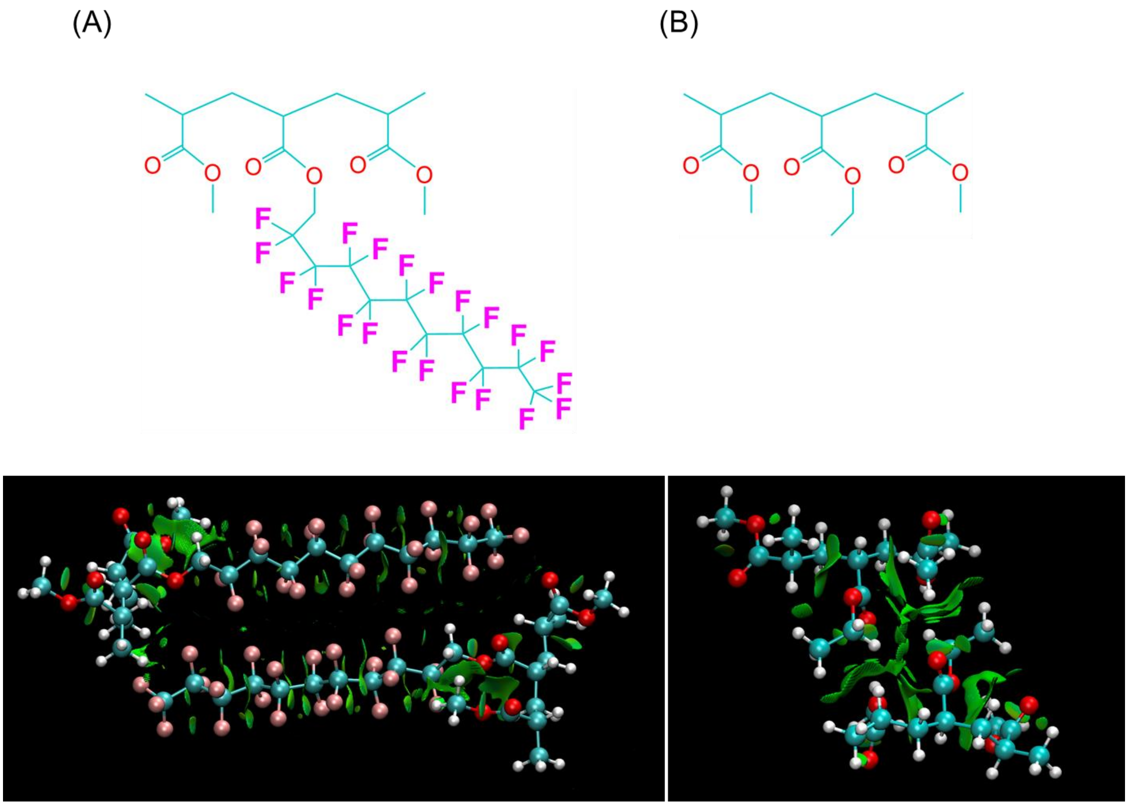

Supplementary Fig. 4. Chemical structures and noncovalent interaction (NCI) images for the density functional theory-optimized (A) fluoroalkyl ester pair and (B) alkyl ester pair compounds. Strong attractive interactions, weak interactions, and strong repulsive interactions are indicated with blue, green, and red regions, respectively, in the 3D-NCI plot. Attractive interactions between alkyl ester compounds are obvious, whereas there is little interaction between fluoroalkyl side chains. The interaction energy for the alkyl ester pair compound was determined to be 45 kJ mol^{-1}, while the interaction energy between the fluoroalkyl side chains in the fluoroalkyl ester pair was only 23 kJ mol^{-1}.

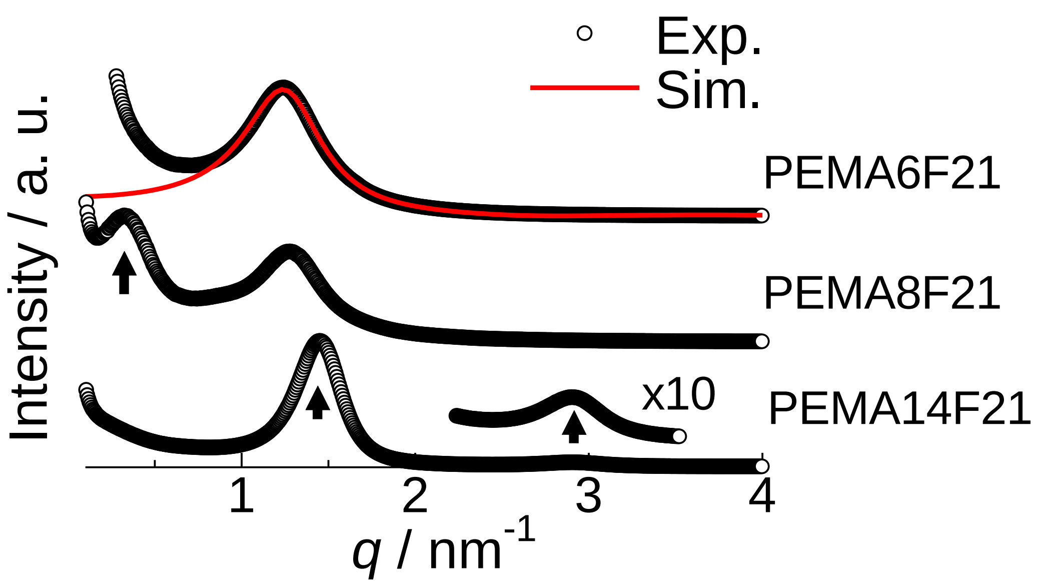

Supplementary Fig. 5. Small-angle X-ray scattering patterns for the indicated samples. The experimental pattern of PEMA6F21 was simulated using the Yarusso–Cooper model.[2] The R_1, R_{CA}, and ND values obtained by the simulation were 1.59 nm, 2.22 nm, and 17.0 (10 nm)$^{-3}$, respectively. Scatterings from the crystalline structures are indicated with arrows.

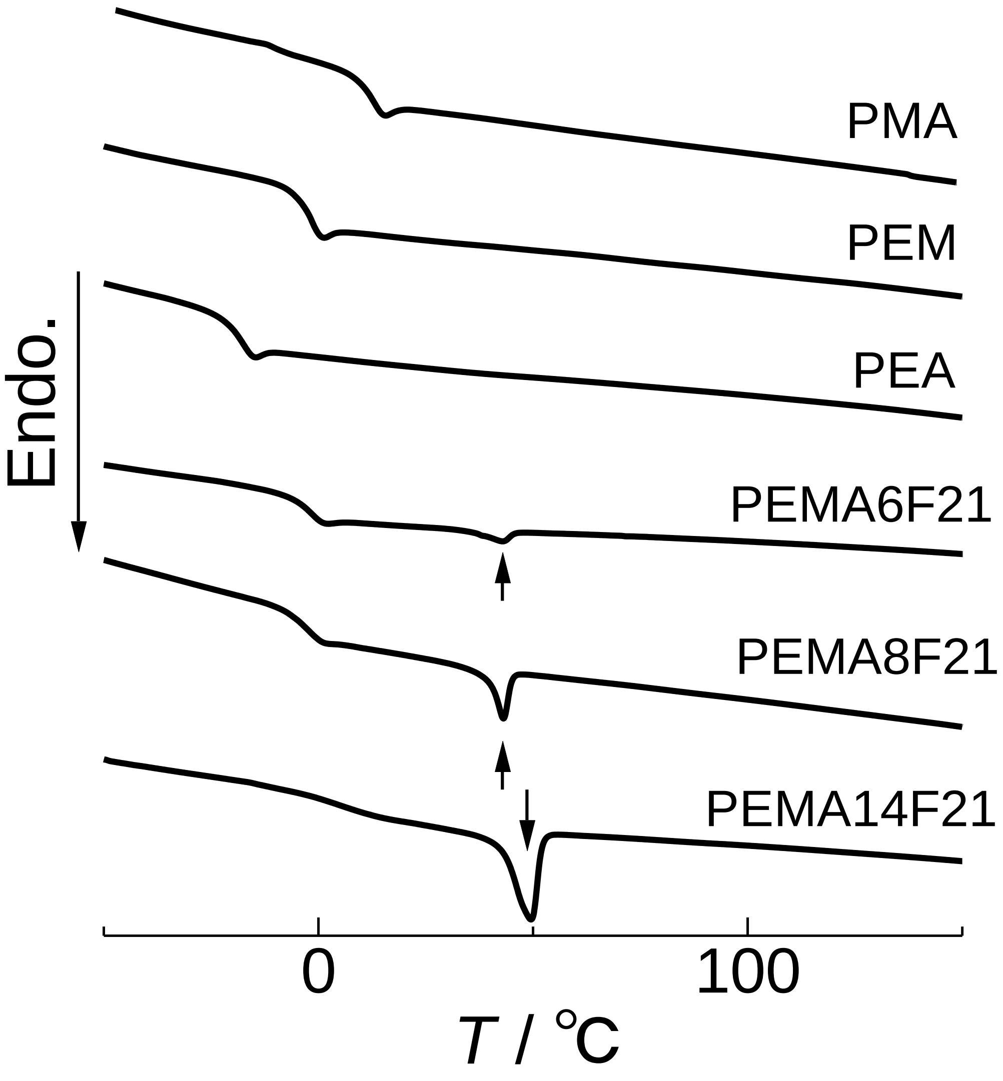

Supplementary Fig. 6. Differential scanning calorimetry traces of the indicated samples heated at 10°C min^{-1}. The melting of the crystalline fluoroalkyl side chain is indicated with an arrow.

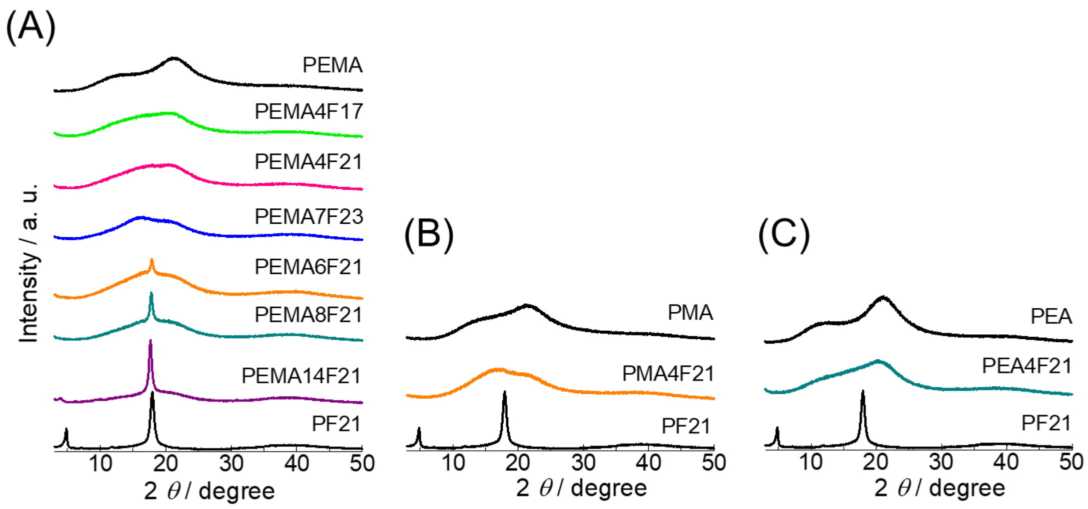

Supplementary Fig. 7. X-ray diffraction patterns of (A) PEMA*x*F*y*, (B) PMA4F21, and (C) PEA4F21. The diffraction from the crystalline component of the fluoroalkyl components of PEMA6F21, PEMA8F21, PEMA14F21, and PF21 can be observed in the corresponding patterns.

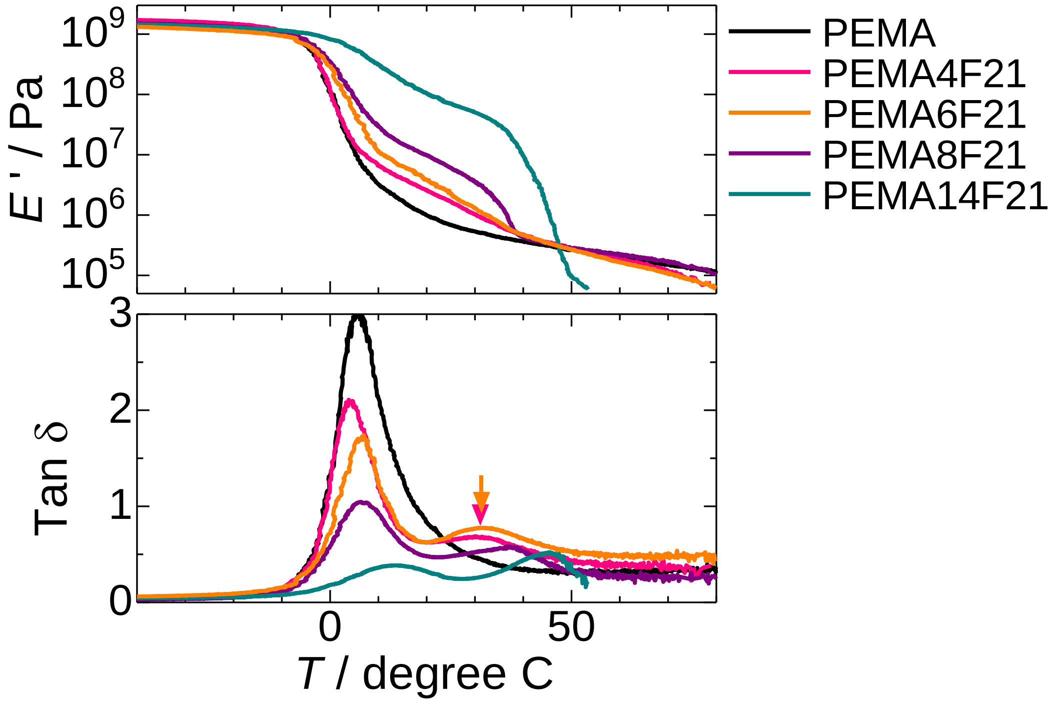

Supplementary Fig. 8. Temperature dependence of storage modulus (*E*') and tanδ at 1 Hz for the indicated samples. The tanδ peak assigned to the network rearrangement is indicated with an arrow. PEMA4F21 and PEMA6F21 having the same fluoroalkyl chain length afforded almost the same peak temperature.

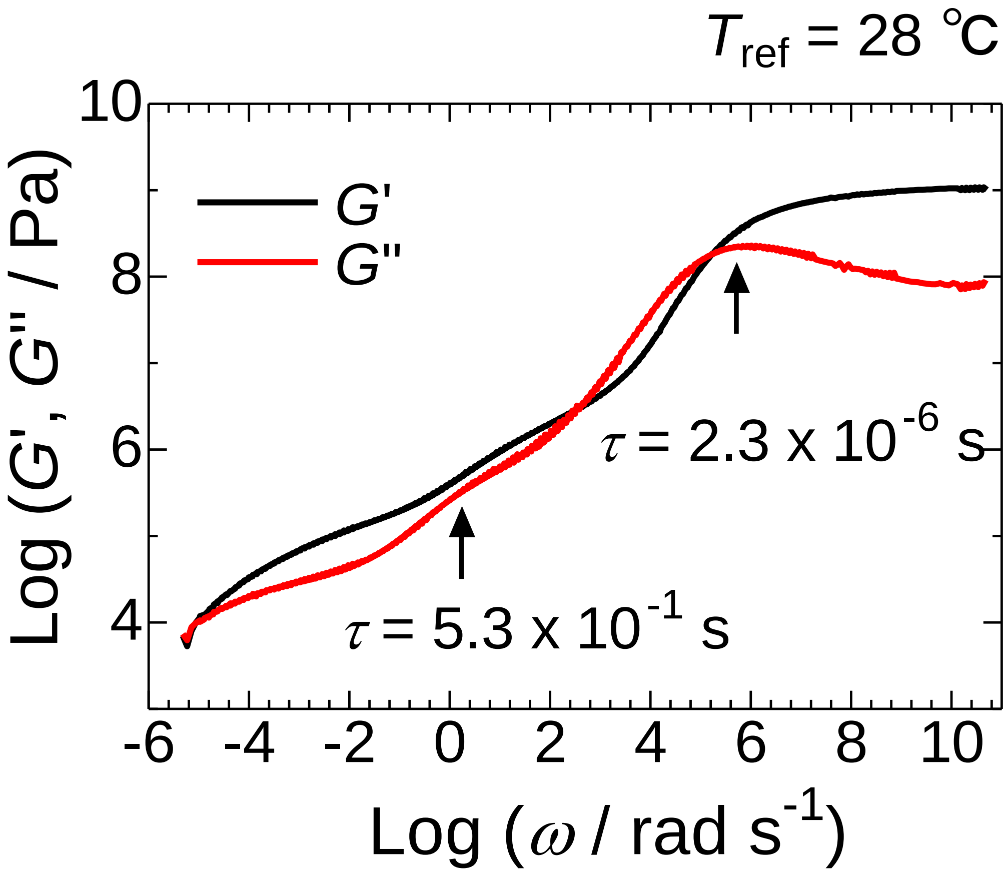

Supplementary Fig. 9. Master curves for storage shear modulus (G') and loss shear modulus (G'') of PEMA4F21 at 28°C. The relaxation times for the segmental motion of the main chain and network rearrangement were 2.3×10^{-6} s and 5.3×10^{-1} s, respectively.

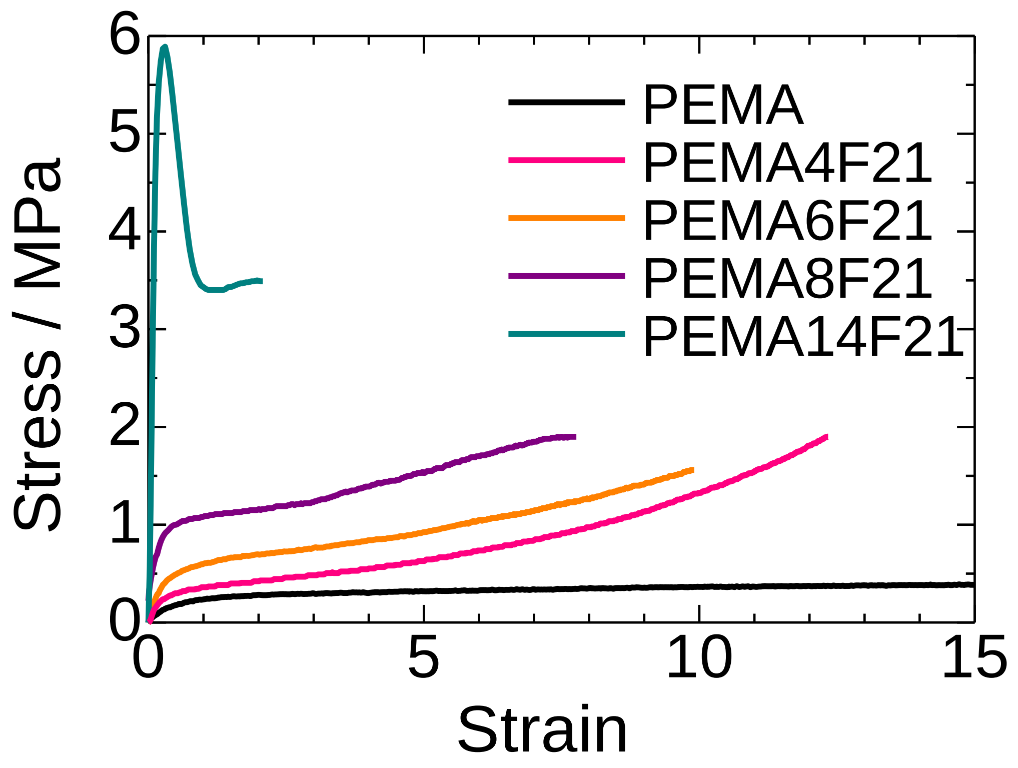

Supplementary Fig. 10. Tensile stress−strain curves for the indicated samples at a strain rate of 0.2 s^{-1} and 27°C ± 1°C.

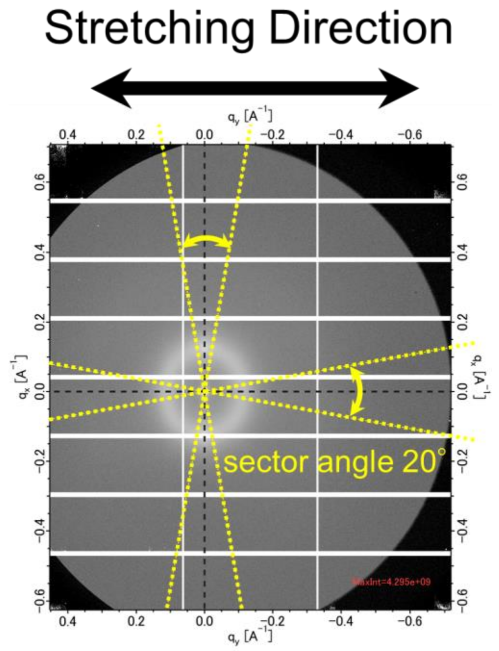

Supplementary Fig. 11. Schematic explanation of the sector averaging of the two-dimensional small-angle X-ray scattering data for meridian and equatorial directions.

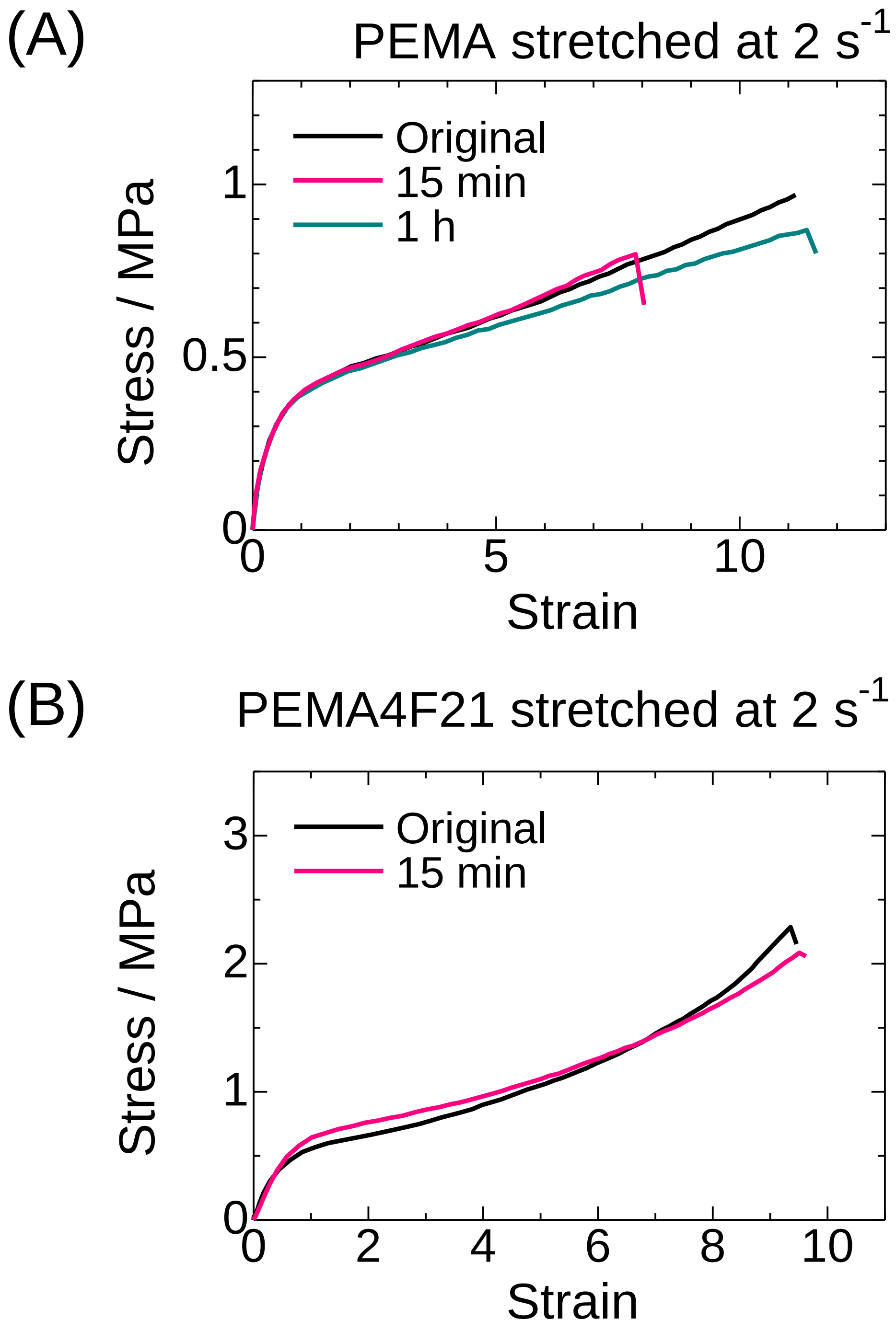

Supplementary Fig. 12. Tensile behaviors of original and self-healed PEMA and PEMA4F21 samples at a strain rate of 2 s^{-1}. (A) The cut PEMA was allowed to self-heal at 27°C ± 1°C for the indicated time period. The healing efficiencies after 15 min and 1 h were 60% ± 20% and 90% ± 20%, respectively. (B) The cut PEMA4F21 was self-healed at 27°C ± 1°C for 15 min. The healing efficiency was 99% ± 9%.

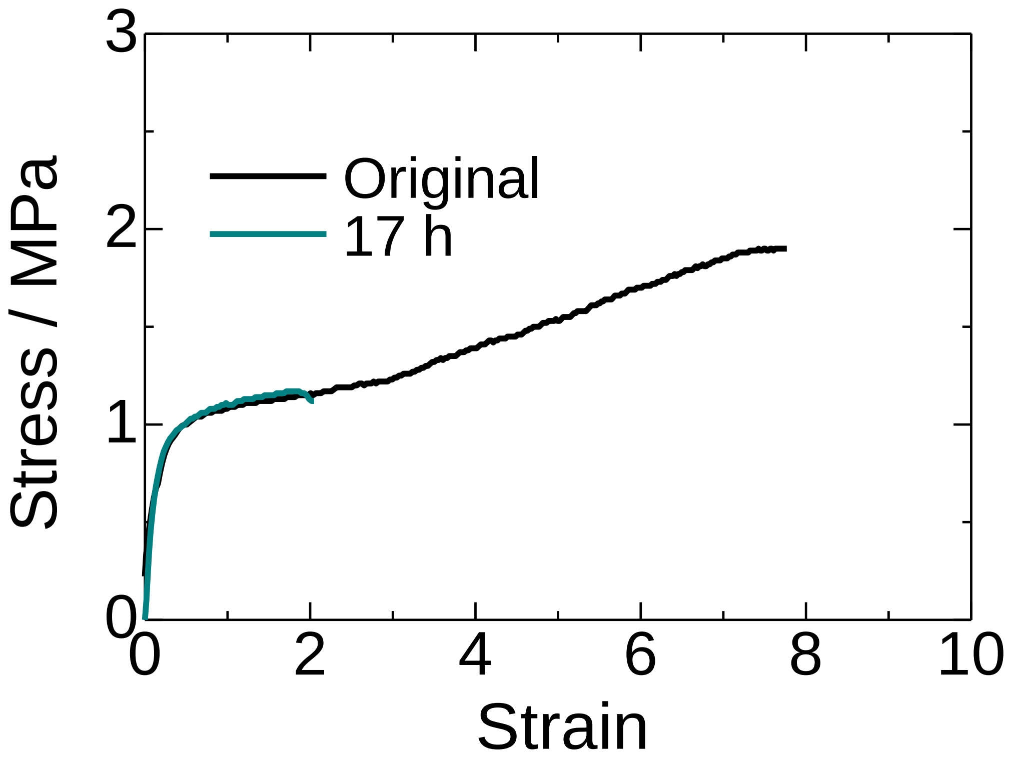

Supplementary Fig. 13. Tensile behavior of original and self-healed PEMA8F21 samples at a strain rate of 0.2 s^{-1}. The cut PEMA8F21 was allowed to self-heal at 27°C ± 1°C for 17 h. The healing efficiency was 23% ± 9%.

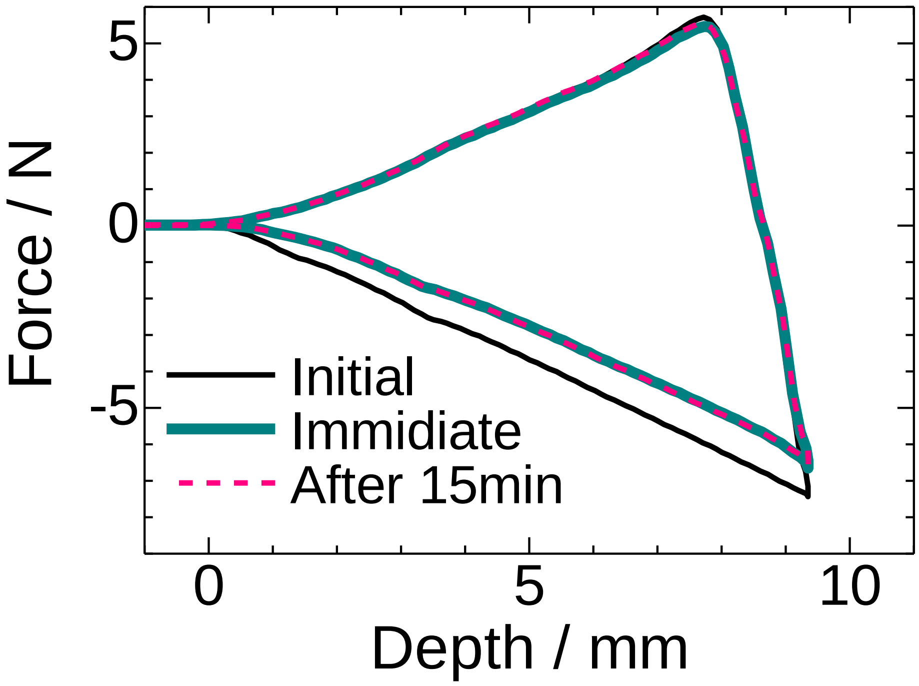

Supplementary Fig. 14. Monitoring of the sticking behavior of a chemically crosslinked silicone elastomer with a needle at a rate of 1 s^{-1} and 27°C ± 1°C. The sticking was repeated immediately and after 15 min. The recovery of the mechanical strength was not observed for the repeated measurement.

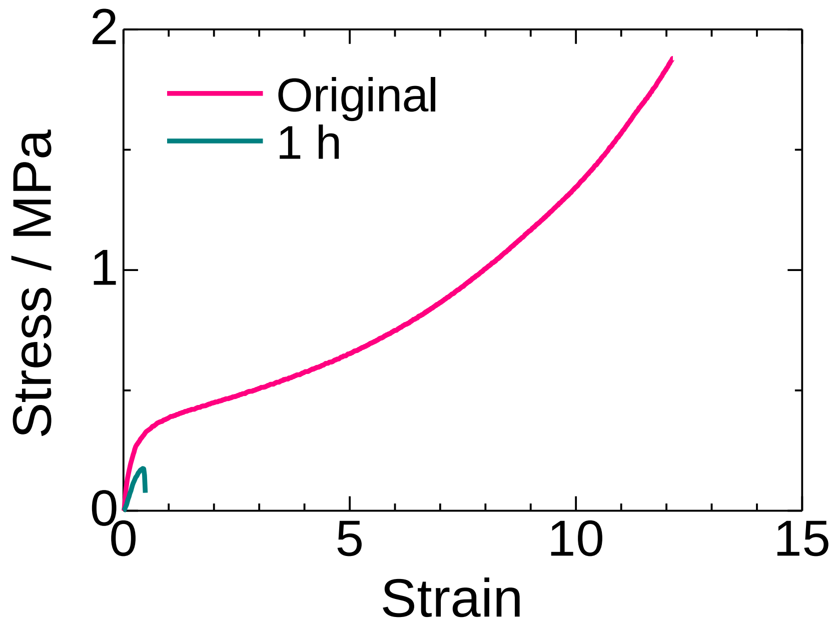

Supplementary Fig. 15. Tensile behavior of PEMA4F21 after contacting the original surfaces at 27°C ± 1°C for 1 h. The specimen was stretched at a strain rate of 0.2 s^{-1}. The data for original PEMA4F21 is also shown for comparison.

3. References

(1) Hasegawa, T., Shimoaka, T., Tanaka, Y., Shinoya, N. & Morita, K. An origin of complicated infrared spectra of perfluoroalkyl compounds involving a normal alkyl group. *Chem. Lett.* **44**, 834–836 (2015).

(2) Yarusso, D. J., & Cooper, S. L. Analysis of SAXS data from ionomer systems. *Polymer* **26**, 371–378 (1985).

(3) Maeda, S., Taketsugu, T., Morokuma, K. & Ohno, K. Anharmonic downward distortion following for automated exploration of quantum chemical potential energy surfaces. *Bull. Chem. Soc. Jpn.*, **87**, 1315–1334 (2014).

(4) Maeda, S., Harabuchi, Y., Takagi, M., Saita, K., Suzuki, K., Ichino, T., Sumiya, Y., Sugiyama, K. & Ono, Y. Implementation and performance of the artificial force induced reaction method in the GRRM17 program. *J. Comput. Chem.*, **39**, 233–251 (2018).

(5) Boys, S. F. & Bernardi, F. The calculation of small molecular interactions by the differences of separate total energies. some procedures with reduced errors. *Mol. Phys.*, **19**, 553–566 (1970).

(6) Boto, R. A., Peccati, F., Laplaza, R., Quan, C., Carbone, A., Piquemal, J. -P., Maday, Y. & Contreras-Garcia, J. *NCIPLOT4: A new step towards a fast quantification of noncovalent interactions*, DOI https://github.com/juliacontrerasgarcia/nciplot.

(7) Johnson, E. R., Keinan, S., Mori-Sanchez, P., Contreras-Garcia, J., Cohen, A. J. & Yang, W. Revealing noncovalent interactions. *J. Am. Chem. Soc.*, **132**, 6498–6506 (2010).

(8) Contreras-Garcia, J., Johnson, E. R., Keinan, S., Chaudret, R., Piquemal, J. -P., Beratan, D. N. & Yang, W. NCIPLOT: a program for plotting non-covalent interaction regions. *J. Chem. Theory Comput.*, **7**, 625–632 (2011).
